# The Malaria TaqMan Array Card Includes 87 Assays for Plasmodium falciparum Drug Resistance, Identification of Species, and Genotyping in a Single Reaction

**DOI:** 10.1128/AAC.00110-17

**Published:** 2017-04-24

**Authors:** Suporn Pholwat, Jie Liu, Suzanne Stroup, Shevin T. Jacob, Patrick Banura, Christopher C. Moore, Fang Huang, Miriam K. Laufer, Eric Houpt, Jennifer L. Guler

**Affiliations:** a Division of Infectious Diseases and International Health, Department of Medicine, University of Virginia, Charlottesville, Virginia, USA; b Department of Medicine, Division of Allergy and Infectious Diseases, University of Washington, Seattle, Washington, USA; c Department of Community Health, Masaka Regional Referral Hospital, Masaka, Uganda; d Division of Malaria Research, Institute for Global Health, University of Maryland School of Medicine, Baltimore, Maryland, USA; e National Institute of Parasitic Diseases, Chinese Center for Disease Control and Prevention, Shanghai, China; f Department of Biology, University of Virginia, Charlottesville, Virginia, USA

**Keywords:** TaqMan PCR, antimalarial resistance, malaria, mutation, surveillance tool

## Abstract

Antimalarial drug resistance exacerbates the global disease burden and complicates eradication efforts. To facilitate the surveillance of resistance markers in countries of malaria endemicity, we developed a suite of TaqMan assays for known resistance markers and compartmentalized them into a single array card (TaqMan array card, TAC). We included 87 assays for species identification, for the detection of Plasmodium falciparum mutations associated with chloroquine, atovaquone, pyrimethamine, sulfadoxine, and artemisinin resistance, and for neutral single nucleotide polymorphism (SNP) genotyping. Assay performance was first optimized using DNA from common laboratory parasite lines and plasmid controls. The limit of detection was 0.1 to 10 pg of DNA and yielded 100% accuracy compared to sequencing. The tool was then evaluated on 87 clinical blood samples from around the world, and the malaria TAC once again achieved 100% accuracy compared to sequencing and in addition detected the presence of mixed infections in clinical samples. With its streamlined protocol and high accuracy, this malaria TAC should be a useful tool for large-scale antimalarial resistance surveillance.

## INTRODUCTION

Despite a decline in incidence, malaria remains an important cause of morbidity and mortality across the world. There were 214 million cases of malaria globally, leading to an estimated 429,000 to 730,500 deaths in 2015 (1, 2). Antimalarial drug resistance has substantial implications for malaria control. The emergence of resistance to chloroquine in the 1980s led to its replacement with sulfadoxine-pyrimethamine (SP) for the treatment of uncomplicated malaria (3). The rapid development of resistance to these drugs then led to the recommendation of artemisinin combination therapy (4). Recently, resistance to artemisinin has been detected in the Greater Mekong subregion including the China-Myanmar border (5–10). Because of this history, monitoring antimalarial resistance is an important component of successful malaria control programs.

Standard methodologies to assess antimalarial sensitivity are time-consuming, technically challenging, and expensive. To estimate clinical efficacy (*in vivo* sensitivity), the response to antimalarial treatment needs to be monitored in the patient for at least 14 days (11). *Ex vivo* assessment of Plasmodium falciparum sensitivity requires tissue culture facilities and fresh blood products for parasite propagation, which are not readily available in countries of malaria endemicity. Genetic markers of resistance have been identified for several clinical antimalarials, and assessment of parasite DNA for these markers is a useful method for resistance detection (12–20). Several molecular tools have been developed to detect these markers, including PCR-restriction fragment length polymorphism (RFLP) (21), high-resolution melt analysis (22), quantitative PCR (23, 24), and loop-mediated isothermal amplification (LAMP) (25). Although accurate, these PCR-based methods are onerous because multiple markers need to be assessed. Luminex-based assays (26, 27), microarrays (28), and whole-genome sequencing of clinical samples (29, 30) can achieve greater throughput but are difficult to implement in field settings. We therefore sought to create a comprehensive, easy-to-perform method to track many resistance markers from multiple samples in a single run. The TaqMan array card (TAC) is a customizable 384-well card that compartmentalizes each sample into 48 different quantitative PCRs. TAC protocols are streamlined (due to several self-contained reagents), and eight patient samples can be run simultaneously. Our group has applied this technology successfully for detection of multiple tuberculosis (TB) drug resistance markers (31, 32), syndromic pathogen detection (33, 34), and pneumococcal serotyping (35) and has found excellent reproducibility between multiple laboratories across Africa, Asia, and America.

Here, we describe the construction and testing of the malaria TAC. This initial card design includes 70 assays for the detection of resistance-associated mutations in the P. falciparum *CRT* [*pfCRT*] chloroquine), *pfCYTB* (atovaquone), *pfDHFR* (pyrimethamine), *pfDHPS* (sulfadoxine), *pfMDR1* (multidrug resistant), and *pfKelch13* (artemisinin) genes. Additionally, assays for the detection of the five Plasmodium species targeting humans (falciparum, vivax, ovale, malariae, and knowlesi) and 12 genotyping single nucleotide polymorphisms (SNPs) were included to provide additional epidemiologic information about the samples.

## RESULTS

### TaqMan assay performance.

**FIG 1:**
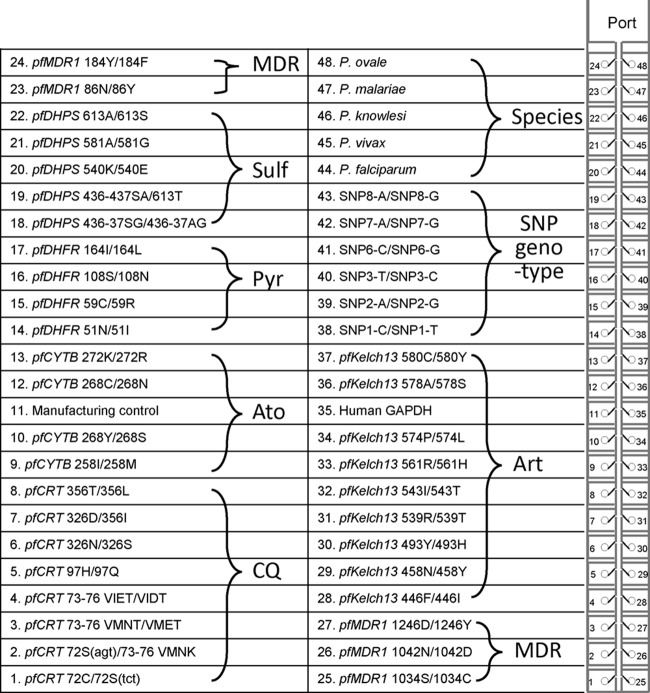
Malaria TAC design. The antimalarial resistance-focused TaqMan array card (malaria TAC) includes eight ports/card (one port is shown), and each port is connected to 48 assay wells. Each assay well contains prespotted primers and probes and is configured as shown on the basis of gene, codon, and wild-type/mutant-specific loci. The antimalarial to which resistance is conferred is listed next to each group of targets (MDR, multidrug resistance; Sulf, sulfadoxine; Pyr, pyrimethamine; Ato, atovaquone; CQ, chloroquine; Art, artemisinin). For genotyping, the SNPs are identified as follows: SNP1, *Pf*_01_000130573; SNP2, *Pf*_01_000539044; SNP3, *Pf*_02_000842803; SNP6, *Pf*_06_000145472; SNP7, *Pf*_06_000937750; SNP8, to *Pf*_07_000277104. Plasmodium species-specific probes are included to confirm the presence of P. falciparum DNA in each sample.

The 35 duplex TaqMan assays for resistance markers, 6 duplex assays for SNP genotyping, and 5 singleplex assays for species identification as well as a control assay for human glyceraldehyde-3-phosphate dehydrogenase (hGAPDH) (Fig. 1) were first tested against 35 other blood pathogens, and no cross-reactivity (detection of false positives) was observed (see Materials and Methods for a list of pathogens) (data not shown). Specificity was then tested against 11 P. falciparum lines, P. vivax, P. knowlesi, human DNA, and 17 synthetic plasmid controls in the 384-well plate format (see Fig. S1 in the supplemental material). For most assays (38/47), we detected no cross-reactivity between any of the duplexed assays (wild-type and mutant loci and major and minor SNP alleles) or among the five Plasmodium species. We observed minor cross-reactivity in 9 of 47 assays (*pfCRT* 73-76VMET, *pfCRT* 326S, *pfCRT* 356I, *pfCYTB* 268Y, *pfCYTB* 268C, *pfDHPS* 613A, *pfMDR1* 1246Y, SNP1-T, and SNP2-G) (Fig. S1). Such cases were observed only when high concentrations of DNA (>1 ng) were used and were eliminated by adjusting the threshold.

Next, the PCR performance of each primer/probe assay was determined on 384-well plates and then later with the TAC format. To test the amplification efficiency of each assay, DNA from either individual parasite lines or plasmid controls was tested in triplicate for six serial dilutions (see Materials and Methods for details). The overall linearity (*R*^2^) of the 88 targets, including hGAPDH, was 0.990 ± 0.01 and 0.994 ± 0.01; PCR efficiencies were 90% ± 6.4% and 92% ± 7.9% for the 384-well plates and TAC formats, respectively (Tables S1 and S2). The limit of detection was similar on both 384-well plates and TaqMan array cards, ranging between 10 to 100 plasmid copies per reaction or 4.03 to 403 copy equivalent genomic DNAs (0.1 to 10 pg) per reaction (Tables S1 and S2).

### Malaria TAC evaluation.

The performance of the TaqMan array card was evaluated using genomic DNA from 18 laboratory parasite lines and 87 clinical samples. The malaria TAC successfully detected the correct wild-type or mutant loci in 99% ± 4.1% of reaction products using 18 laboratory parasite strains; those that were left undetermined represented parasite lines with mutations other than those included in malaria TAC (Table 1, *pfCRT* 97 and *pfDHPS* 436–437). The malaria TAC successfully detected either wild-type or mutant loci for 89% ± 7.3% of reactions using 87 clinical samples (Table 1). The *pfCRT* 72-76, *pfCRT* 97, *pfDHFR* 164, and *pfDHPS* 540 targets had the lowest rates of detection in this set of samples (<80%) (Table 1). The undetermined samples were mostly from the Malawi repository, with an average detection level of 78% ± 9.0%. Thailand-, China-, and Uganda-derived samples exhibited detection levels of 95% ± 5.3%, 90% ± 18.4%, and 100%, respectively (Table S3). The malaria TAC successfully detected two clinical samples that contained non-P. falciparum parasite DNA, including one each of P. vivax and P. ovale (Table 1).

**TABLE 1:** Comparison of malaria TAC performance using laboratory parasites and clinical samples

Target	TAC performance on culture parasite lines (*n* = 18)		TAC performance on clinical samples (*n* = 87)	
	No. (%) of positive parasite lines (*n* = 18)	Mean *C_T_* ± SD^*a*^	No. (%) of positive clinical samples (*n* = 87)	Mean *C_T_* ± SD
Human *GAPDH*			80 (92)	30.7 ± 3.4
P. vivax	1 (100)	24.2	1 (100)	29.2
P. knowlesi	1 (100)	19.4		
P. ovale			1 (100)	29.5
P. falciparum	16 (100)	21.9 ± 3.0	81 (95)	29.4 ± 5.4
*pfCRT* 72–76	16 (100)	24.4 ± 2.6	62 (73)	34.7 ± 4.3
*pfCRT* 97	15 (94)^*b*^	25.9 ± 2.7	56 (66)	37.6 ± 4.6
*pfCRT* 326	16 (100)	23.3 ± 2.9	80 (94)	32.0 ± 4.9
*pfCRT* 356	16 (100)	24.0 ± 3.3	80 (94)	31.7 ± 5.1
*pfCYTB* 258	16 (100)	18.5 ± 2.4	85 (100)	25.2 ± 4.6
*pfCYTB* 268	16 (100)	18.1 ± 2.5	81 (95)	26.6 ± 4.1
*pfCYTB* 272	16 (100)	19.9 ± 2.4	83 (98)	27.6 ± 4.5
*pfDHFR* 51	16 (100)	25.9 ± 2.8	72 (85)	35.7 ± 4.1
*pfDHFR* 59	16 (100)	23.8 ± 2.5	76 (89)	33.4 ± 4.8
*pfDHFR* 108	16 (100)	25.6 ± 3.1	70 (82)	36.0 ± 4.2
*pfDHFR* 164	16 (100)	23.2 ± 2.5	66 (78)	33.9 ± 4.6
*pfDHPS* 436–437	12 (75)^*c*^	24.1 ± 3.4	79 (93)	30.8 ± 4.5
*pfDHPS* 540	16 (100)	25.8 ± 3.5	61 (72)	36.1 ± 4.4
*pfDHPS* 581	16 (100)	24.5 ± 3.3	73 (86)	32.5 ± 5.2
*pfDHPS* 613	16 (100)	23.2 ± 3.1	77 (91)	30.2 ± 4.8
*pfMDR1* 86	16 (100)	23.9 ± 3.2	78 (92)	32.2 ± 4.3
*pfMDR1* 184	16 (100)	24.2 ± 3.1	74 (87)	33.3 ± 4.4
*pfMDR1* 1034	16 (100)	22.4 ± 3.2	74 (87)	32.7 ± 5.1
*pfMDR1* 1042	16 (100)	22.5 ± 3.0	74 (87)	33.4 ± 4.4
*pfMDR1* 1246	16 (100)	24.8 ± 3.1	75 (88)	34.5 ± 4.7
*pfKelch13* 446	16 (100)	22.2 ± 2.9	76 (89)	31.3 ± 4.8
*pfKelch13* 458	16 (100)	25.0 ± 2.7	77 (91)	34.3 ± 4.6
*pfKelch13* 493	16 (100)	23.8 ± 3.0	78 (92)	31.8 ± 4.6
*pfKelch13* 539	16 (100)	24.7 ± 2.9	77 (91)	33.5 ± 4.3
*pfKelch13* 543	16 (100)	21.8 ± 2.8	74 (87)	29.4 ± 4.6
*pfKelch13* 561	16 (100)	22.2 ± 2.8	79 (93)	29.6 ± 4.6
*pfKelch13* 574	16 (100)	22.4 ± 2.8	78 (92)	29.7 ± 4.6
*pfKelch13* 578	16 (100)	23.3 ± 2.8	76 (89)	30.3 ± 4.7
*pfKelch13* 580	16 (100)	24.5 ± 3.1	78 (92)	31.7 ± 5.1
*Pf*_01_000130573	16 (100)	25.0 ± 2.8	81 (95)	31.6 ± 5.0
*Pf*_01_000539044	16 (100)	26.3 ± 3.0	73 (86)	34.1 ± 5.4
*Pf*_02_000842803	16 (100)	25.5 ± 3.1	74 (87)	32.0 ± 5.3
*Pf*_06_000145472	16 (100)	23.3 ± 2.9	76 (89)	30.2 ± 4.7
*Pf*_06_000937750	16 (100)	23.9 ± 3.2	76 (89)	32.9 ± 4.4
*Pf*_07_000277104	16 (100)	24.5 ± 3.0	75 (88)	32.2 ± 4.5
Mean % positive (±SD)	(99.2 ± 4.1)		(89 ± 7.3)	

a *C_T_*, cycle threshold.

b One line was negative with both the wild-type probe (H) and mutant probe (Q) by TAC; sequencing mutant (L).

c Four lines were negative with both the wild-type probe (SG) and mutant probe (AG or SA); sequencing mutant (FG).

The allelic distribution of the loci successfully detected by the malaria TAC is shown in Fig. S2. The majority of clinical samples showed *pfCRT* mutations, except for those from Malawi. All samples displayed *pfDHFR* and *pfDHPS* mutations, but none were observed in *pfCYTB*. The number and type of *pfMDR1* and *pfKelch13* mutations varied depending on the country of origin. The pattern of P. falciparum mutant alleles detected in our analysis is summarized in Table S4. Overall, we detected 13 distinct resistance marker genotypes from 16 laboratory parasite lines (data not shown). This estimation was more difficult for clinical samples since some targets were not detectable and therefore yielded incomplete data. However, from clinical samples in which all drug resistance loci were detected, the malaria TAC successfully detected 13 distinct resistance marker genotypes or antibiograms (data not shown).

For validation, Sanger sequencing was performed in parallel on all laboratory parasites and ∼50% of clinical parasites. Results from the malaria TAC showed 100% sensitivity, specificity, and accuracy (Table 2). One laboratory parasite sample was negative for both wild-type (H) and mutant (Q) probes by malaria TAC at *pfCRT* 97. Sequencing revealed that this sample harbored an alternative allele that was not tested in our current design (H97L) (Tables 1 and 2). Similarly, four *pfDHPS* 436–437 mutants had an alternative allele (FG) that was not covered by the malaria TAC and was therefore not detected with the wild-type (SG) or mutant (AG or SA) probe (Tables 1 and 2).

**TABLE 2:** Comparison of malaria TAC and Sanger sequencing results^*a*^

Target	TAC probe	Sequencing result		TAC sensitivity (%)	TAC specificity (%)	TAC accuracy (%)
		Mutant	Wild type			
*pfCRT* 72–76	Mutant	42	0	100	100	100
	Wild type	0	16			
*pfCRT* 97	Mutant	1^*b*^	0	100	100	100
	Wild type	0	57			
*pfCRT 326*	Mutant	36	0	100	100	100
	Wild type	0	22			
*pfCRT 356*	Mutant	35	0	100	100	100
	Wild type	0	23			
*pfCYTB* 258	Mutant	0	0	N/A^*d*^	100	100
	Wild type	0	58			
*pfCYTB* 268	Mutant	1	0	100	100	100
	Wild type	0	57			
*pfCYTB* 272	Mutant	0	0	N/A	100	100
	Wild type	0	58			
*pfDHFR* 51	Mutant	49	0	100	100	100
	Wild type	0	9			
*pfDHFR* 59	Mutant	53	0	100	100	100
	Wild type	0	5			
*pfDHFR* 108	Mutant	56	0	100	N/A	100
	Wild type	0	2			
*pfDHFR 164*	Mutant	27	0	100	100	100
	Wild type	0	31			
*pfDHPS* 436/37	Mutant	13^*c*^	0	100	100	100
	Wild type	0	45			
*pfDHPS* 540	Mutant	48	0	100	N/A	100
	Wild type	0	10			
*pfDHPS* 581	Mutant	30	0	100	100	100
	Wild type	0	28			
*pfDHPS* 613	Mutant	4	0	100	100	100
	Wild type	0	54			
*pfMDR1* 86	Mutant	10	0	100	100	100
	Wild type	0	48			
*pfMDR1* 184	Mutant	25	0	100	100	100
	Wild type	0	33			
*pfMDR1* 1034	Mutant	2	0	100	100	100
	Wild type	0	56			
*pfMDR1* 1042	Mutant	5	0	100	100	100
	Wild type	0	53			
*pfMDR1* 1246	Mutant	6	0	100	100	100
	Wild type	0	52			
*pfKelch13* 446	Mutant	15	0	100	100	100
	Wild type	0	43			
*pfKelch13* 458	Mutant	0	0	N/A	100	100
	Wild type	0	58			
*pfKelch13* 493	Mutant	2	0	100	100	100
	Wild type	0	56			
*pfKelch13* 539	Mutant	1	0	100	100	100
	Wild type	0	57			
*pfKelch13* 543	Mutant	1	0	100	100	100
	Wild type	0	57			
*pfKelch13* 561	Mutant	1	0	100	100	100
	Wild type	0	57			
*pfKelch13* 574	Mutant	3	0	100	100	100
	Wild type	0	55			
*pfKelch13* 578	Mutant	0	0	N/A	100	100
	Wild type	0	58			
*pfKelch13* 580	Mutant	2	0	100	100	100
	Wild type	0	56			
		**Minor allele**	**Major allele**			
*Pf*_01_000130573	Minor allele (T)	25	0	100	100	100
	Major allele (C)	0	33			
*Pf*_01_000539044	Minor allele (G)	12	0	100	100	100
	Major allele (A)	0	46			
*Pf*_02_000842803	Minor allele (C)	33	0	100	100	100
	Major allele (T)	0	25			
*Pf*_06_000145472	Minor allele (G)	21	0	100	100	100
	Major allele (C)	0	37			
*Pf*_06_000937750	Minor allele (G)	28	0	100	100	100
	Major allele (A)	0	30			
*Pf*_07_000277104	Minor allele (G)	34	0	100	100	100
	Major allele (A)	0	24			

a Data represent both laboratory parasites (*n = 16) and clinical samples (n* = 42).

b Negative with both the wild-type probe (H) and mutant probe (Q) by TAC; sequencing mutant (L).

c Four of 13 samples were negative with both the wild-type probe (SG) and mutant probe (AG or SA); sequencing mutant (FG).

d N/A, not applicable.

**FIG 2:**
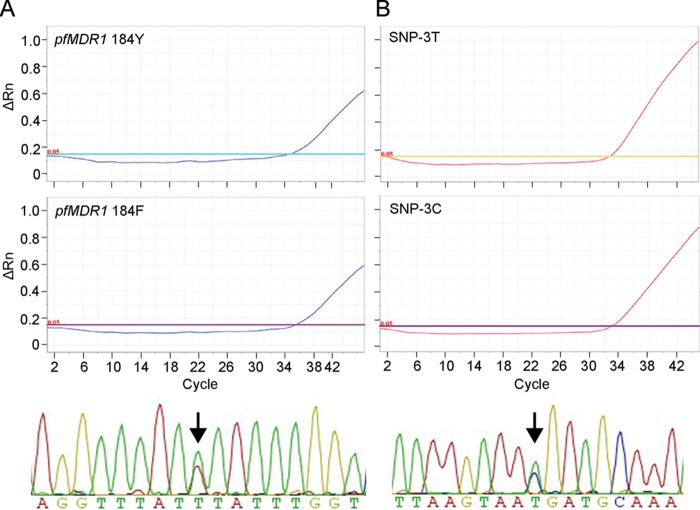
Examples of heteroresistance and mixed infection as determined by the malaria TAC. The DB133 and DB009 samples were detected as mixed alleles by the malaria TAC and then sequence confirmed. (A) TaqMan probe-based amplification plots are shown for the wild-type probe (*pfMDR1* 184Y) and mutant probe (*pfMDR1* 184F); Sanger sequencing shows mixed T/A residues (arrow), indicating mixed F(TTT) and Y(TAT) alleles for the DB133 sample (bottom panel). (B) TaqMan probe-based amplification plots are shown for the major and minor allele probes for SNP3 (*Pf*_02_000842803 (T) and *Pf*_02_000842803 (C); Sanger sequencing shows mixed T/C residues for the DB009 sample (arrow).

During the assessment of clinical parasite genotypes, we found that 7/85 (8%) clinical DNA samples were positive for multiple probes at a single locus (termed hetero-resistance [data not shown]). These findings were originally detected using the malaria TAC but were subsequently sequence confirmed (Fig. 2 and S3). For example, DB133 was positive for both the wild-type (184Y) and mutant (184F) *pfMDR1* probes; Sanger sequencing showed mixed T/A as position 184, which indicated the presence of both F(TTT) and Y(TAT) (Fig. 2).

Due to space limitations, only 6 of 24 original genotyping SNPs (36) could be included in this initial malaria TAC design. With this limited set, we detected 12 distinct barcodes from 16 laboratory parasite lines (data not shown) and 67 distinct barcodes from 85 clinical samples (Table 3) (18 samples exhibited incomplete data and were not included in this analysis). The set of distinct barcodes from clinical samples included 13 shared and 14 unique barcodes. Four of the 13 shared barcodes were found in multiple samples from the same country, 8/13 were shared across two different countries, and 1/13 (CATCAG) was found in all four countries. We also detected 14 (21%) barcodes that were unique and contained mixed alleles, which were also confirmed by sequencing (Fig. 2 and S4). In one example, sample DB009 was positive for both the major and minor allele probes at SNP3 (*Pf*_02_000842803 T and C, respectively); Sanger sequencing also showed a mixed T/C at this position (Fig. 2).

**TABLE 3:**
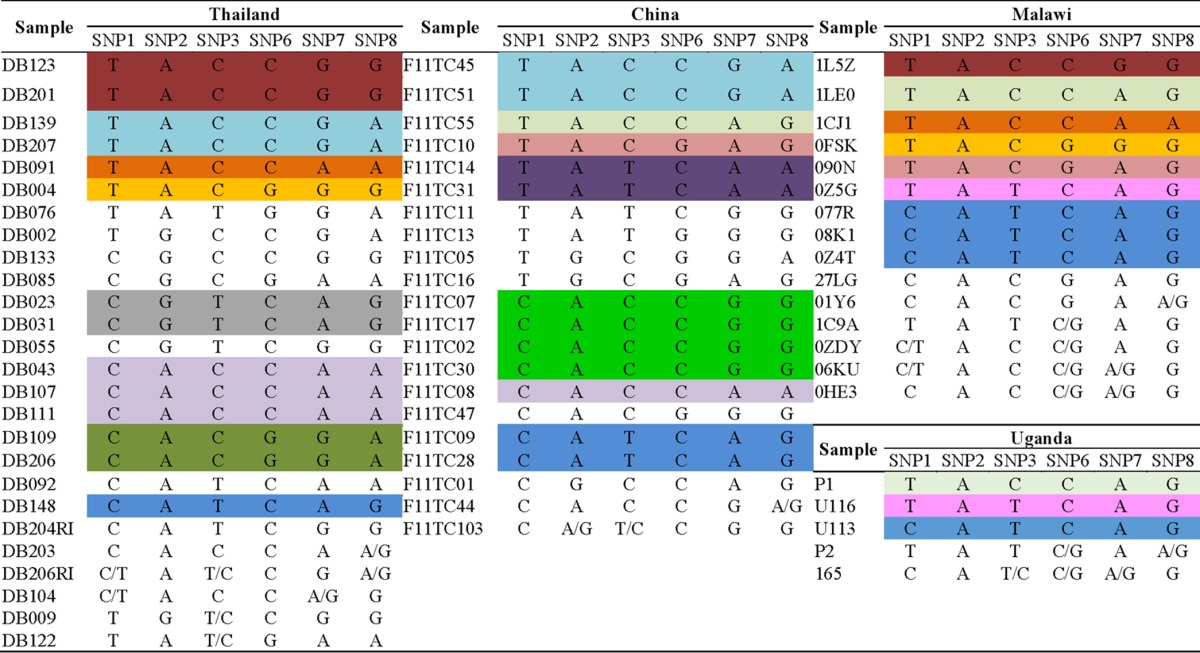
SNP barcode profile of 67 clinical samples^*a*^

a Nonunique haplotypes are highlighted in the same color. Slashes indicate mixed strains (e.g., A/G). SNP1, *Pf_01_000130573; SNP2, Pf*_01_000539044; SNP3, *Pf*_02_000842803; SNP6, *Pf*_06_000145472; SNP7, *Pf*_06_000937750; SNP8, *Pf*_07_000277104.

## DISCUSSION

In this study, we developed and tested a quantitative PCR-based TaqMan array card to detect the majority of known antimalarial resistance-associated mutations. The malaria TAC yielded excellent specificity, with no cross-reactivity to other pathogens or human genes and no unexplained cross-reactivity between alleles (Fig. S1 in the supplemental material; also data not shown). This tool displayed excellent sensitivity and accuracy when clonal laboratory parasites were the source of DNA and results were compared with results of Sanger sequencing (Tables 1 and 2). Additionally, the malaria TAC performed well when clinical samples were used (Tables 1 and 2), despite the presence of human DNA (∼80 copies/reaction volume or 4.0 × 10^4^ copies/100 μl of DNA). Based on the detection of heteroresistance and mixed genotypes, this tool has the ability to detect mixed infections (Table 3 and Fig. 2, S3, and S4), which are common, but underappreciated, in clinical samples.

Despite many benefits, there are also some obvious limitations of the malaria TAC. Although future malaria TAC designs are customizable, we are limited to the detection of known loci. For the most part, mutations that contribute to clinical resistance are well known, but new mutations whose significance remains unknown are still being discovered (37). Design flexibility is particularly relevant for emerging artemisinin resistance where novel Kelch13 resistance mutations are being detected (38). As is, our initial design of the malaria TAC appears to be a good approximation of important mutations. For example, we detected only two alleles in parasite DNA that were not included in the initial design (*pfCRT* 97L and *pfDHPS* 436F) (Table 1). These mutations were not originally represented because their global minor allele frequencies (MAF) were well below those for other alleles of these loci (39). Second, we detected ample diversity across the malaria TAC assays: (i) SNP barcodes were covered by 75% of laboratory parasite lines and 61% of clinical samples, and (ii) resistance markers were covered by 81% of laboratory parasite lines and 38% of clinical samples.

From a genotyping standpoint, we were limited in the number of barcodes that could be interrogated. From just five loci, we detected distinct barcodes in ∼61% (41/67) of clinical samples. Since ∼19% of the barcodes were shared and since the majority of these were detected in at least two different countries, it is likely that an expansion of the number of barcodes tested would have revealed that many of these were indeed unique. For example, sample DB148 from Thailand had a genotyping barcode that was shared with two samples from China, three samples from Malawi, and one sample from Uganda. The malaria TAC also detected heteroresistance in this sample at two loci, indicating that it was likely a mixed infection. We propose that expansion of the number of SNP barcodes would have revealed a unique/mixed barcode in this sample that originated from Thailand. Future malaria TAC designs can augment this aspect of assay design.

A challenge to clinical infections is the detection of minor loci from mixed infections. Using the malaria TAC, we detected mixed infections in samples from all four countries. If we take into account both genotyping SNPs and observations of heteroresistance, Thailand exhibited ∼31%, China exhibited ∼10%, Malawi exhibited ∼33%, and Uganda exhibited ∼40% mixed infections. Of course, these were convenience samples that were not systematically collected for purposes of examining heteroresistance, so the absolute numbers are likely not representative of those regions. Four of seven cases of heteroresistance were also detected as mixed infections by genotyping SNPs, indicating that it is possible to accurately detect multiple alleles using the malaria TAC (down to ∼10% [data not shown]). Future assessment of the malaria TAC will work to accurately determine the limit of detection for rare alleles.

Clinical samples also exhibit various levels of parasite densities and thus parasite DNA. Measuring the limit of detection, or LOD, allows the estimation and comparison of sensitivity for various methods. Most of the malaria TAC assays displayed an LOD of 40.3 copies/reaction volume (∼8 to 40 parasites/μl, if single-copy genes were assessed on either the plate or TAC format). The malaria TAC LOD is similar to LODs of other TACs that we have developed (35). Additionally, the LOD is within range of other sensitive genotyping methods used in the malaria field (10 parasites/μl) (24, 40). Future work will explore the malaria TAC limit of detection using clinical samples.

One related concern that was revealed by these studies was the decrease in malaria TAC sensitivity when DNA derived from clinical parasite lines was used. This result is likely a limitation of the quality and quantity of the clinical samples that were used for this analysis. First, the amount of parasite-derived genomic DNA in these samples is unknown because of the presence of an abundance of human blood cell DNA. In order to correct for this, the maximum allowable sample volume was used when the clinical samples were used (20 to 50 μl of a 100-μl reaction mixture). Second, the format and age of the clinical samples varied, which could directly contribute to assay performance (see Materials and Methods for details). For example, the dried blood spots, remarkably, were collected 11 years ago in Thailand. Room temperature storage over long periods of time likely leads to some level of DNA degradation. Evidence for this was observed by the failure of amplification of long PCR product sizes suitable for Sanger sequencing (data not shown). Additionally, most of the undetectable samples were from Malawi; these purified DNA samples had the longest period (3 to 4 years) with an unknown number of freeze-thaw cycles. The cycle thresholds (*C_T_*s) of all malaria TAC assays provided evidence for a small amount/low quality of DNA in the Thailand and Malawi samples; for the hGAPDH assay, Uganda and China samples displayed average *C_T_*s of ∼25 and 28, respectively (collected in 2008 to 2009 and 2016), while the Thailand (2005) and Malawi (2012 to 2013) samples had average *C_T_*s of ∼32 and 34, respectively. These data indicate that freshly collected and prepared DNA would improve sensitivity of the malaria TAC.

While most assays on the malaria TAC performed very well, a few of the assays were problematic. *pfCRT* 72–76, *pfCRT* 97, *pfDHFR* 164, and *pfDHPS* 540 assays appeared suboptimal based on a sensitivity of <80% when they were tested with DNA from clinical parasites (Table 1). Three of these assays were the most challenging to design and exhibited the highest limits of detection. Conversely, *pfCRT* 97 performed well using laboratory parasite-derived DNA and exhibited an LOD similar to that of most of the other assays. It is possible that the presence of human DNA in clinical samples affects the performance of this assay. Although we did not specifically test the *pfCRT* 97 assay, we investigated this possibility by running five randomly chosen duplex assays in the presence and absence of human DNA at multiple ratios. Overall, we did not detect statistically significant differences during this analysis (data not shown). These results indicate that the presence of human DNA in clinical samples likely does not have an impact on these assays, but there is room for future work to investigate this further.

Although not the main purpose of this study, the malaria TAC detected a number of different resistance markers from several clinical isolates. For the most part, our observations are consistent with what has been observed previously. First, we did not detect *pfCRT* mutations from Malawi samples, consistent with the return of chloroquine-susceptible malaria in Malawi after chloroquine use was abandoned (41). The *pfDHFR-pfDHPS* mutant patterns IRNL-SGEGA and IRNL-AGEAA (mutations are underlined), which are linked to high-level sulfadoxine-pyrimethamine (SP) resistance (42), were observed as predominant alleles in Thailand and China samples, respectively. The *pfMDR1* Y184F was the most common mutation observed in this study and has been reported as a common mutation found in Asia and Africa (43–45). Last, we found that 83% of *pfKelch13* alleles from China harbored the F446I mutation; this is the predominant Kelch13 mutation associated with delayed clearance of parasites in patients close to the China-Myanmar border (9, 10).

Overall, our numbers of clinical samples from each country were small, and thus the sensitivity and specificity estimates of the malaria TAC are an approximation. It will be important to further evaluate this tool with additional clinical samples to fully explore its use for surveillance. Based on this pilot study of the malaria TAC, future design alterations could include more genotyping loci (perhaps a new format), removal of cytochrome *b* mutations, and alteration of Kelch13 loci to better detect newly emerging resistance. Additionally, we can replace/redesign suboptimal assays (*pfCRT* 72–76, *pfCRT* 97, *pfDHFR* 164, and *pfDHPS* 540). Deployment of this molecular diagnostic technology requires an expensive real-time PCR platform (Viia7; Applied Biosystems); however, these instruments are already present in a number of countries of malaria endemicity, and this enables testing in country. We are hopeful that the malaria TAC will accelerate the ability to track antimalarial-resistant populations and impact local and national treatment recommendations.

## MATERIALS AND METHODS

### Parasite and human DNA.

DNA from 16 P. falciparum lines with various mutations was used as controls in this study. Genomic DNA was directly obtained from BEI Resources (Manassas, VA, USA): P. falciparum 3D7 (MRA-102G), GB4 (MRA-925G), HB3 (MRA-155G), Dd2 (MRA-150G), 7G8 (MRA-152G), V1/S (MRA-176G), K1 (MRA-159G), TM90C6B (MRA-205G), W2 (MRA-157G), IPC 3445 (MRA-1236G), IPC 4884 (MRA-1238G), and 7C46 (MRA-172G). Cryopreserved parasites originally isolated from Battambang (MRA-1240 [BAT]), Mondolkiri (MRA-1241 [MON]), Pailin (MRA-1236 [PAIL]), and Pursat (MRA-1238 [PUR]), Cambodia, were also obtained from this source, grown in our laboratory, and extracted for DNA as performed previously (46). Two non-P. falciparum species were also obtained for use as controls: genomic DNA of P. knowlesi (MRA-456G; BEI Resources) and cryopreserved P. vivax parasites (MRA-383; BEI Resources) from which DNA was directly extracted. Human control DNA was extracted from whole blood of healthy volunteers in our laboratory. All work was reviewed and approved by Institutional Biosafety and Human Investigation Committees at the University of Virginia.

### Plasmid controls.

Synthetic positive-control plasmids were constructed to cover resistance markers that were not represented in the above listed parasite lines (Genewiz, Inc., South Plainfield, NJ, USA). These loci included pCRT72StctVIET, pCRT72StctVMNT, pCRT73–76VMNT, pCRT73–76VMET, pCRT73–76VIDT, pCRT97Q, pCYTB258M, pCYTB268C, pCYTB268N, pCYTB272R, pKelch13-446I, pKelch13-458Y, pKelch13-561H, pKelch13-574L, pKelch13-578S, and pPlasmepsin4 of P. malariae and P. ovale. A synthetic positive-control plasmid was also constructed to contain the primer and probe regions of all 88 targets and used as a positive control for each malaria TAC run (Genewiz, Inc., South Plainfield, NJ, USA).

### Blood pathogens.

We tested the specificity of our malaria-specific assays against 35 other blood pathogens. Genomic DNAs from 11 pathogens were obtained from BEI Resources (Manassas, VA, USA), as follows: Acinetobacter baumannii (NR-10146), Brucella abortus (NR-2530), Haemophilus influenzae (DD-204), Klebsiella pneumonia (NR-15465), Listeria monocytogenes (NR-13342), Pseudomonas aeruginosa (DD-723), Rickettsia sibirica (NR-10486), Staphylococcus aureus (HM-466D), Streptococcus pneumoniae (HM-145D), Toxoplasma gondii (NR-33509), and Yersinia pestis (NR-2720). Genomic DNAs for 15 pathogens were obtained from ATCC (Manassas, VA, USA), as follows: Bartonella bacilliformis (ATCC 35685D-5), Candida albicans (ATCC 10231D-5), Cryptococcus neoformans (ATCC 66031D-5), Leptospira interrogans (ATCC BAA-1198D-5), Leishmania infantum (ATCC 50134D), Neisseria meningitidis (ATCC 53415D-5), Streptococcus agalactiae (ATCC BAA-611D-5), Streptococcus pyogenes (ATCC 700294D-5), Enterococcus faecium (ATCC 51559D-5), Salmonella enterica serovar Typhi (ATCC 700931D-5), Staphylococcus epidermidis (ATCC 12228D-5), cytomegalovirus (ATCC VR-538D), Epstein-Barr virus (ATCC VR-3247SD), herpes simplex virus 1 (ATCC VR-539DQ), and herpes simplex virus 2 (ATCC VR-540D). Genomic DNAs from nine Mycobacterium species were obtained from our collaborator (Department of Medicine Siriraj Hospital, Mahidol University, Bangkok, Thailand): M. tuberculosis ATCC 27294, M. avium ATCC 700898, M. intracellulare ATCC 13950, M. kansasii ATCC 12478, M. simiae ATCC 25275, M. sherrisii S53, M. abscessus, M. fortuitum ATCC 6841, *and M. peregrinum* ATCC 700686.

### Clinical samples and extractions.

Eighty-seven deidentified clinical samples were used to test the specificity of the malaria TAC. These included samples from Thailand (material, dried blood spots; collected 2005, *n* = 32), China (material, purified DNA; collected 2016, *n* = 21 [47]), Malawi (material, purified DNA; collected 2012 to 2013, *n* = 28), and Uganda (material, blood pellet or purified DNA; collected 2016, *n* = 1 [University of Virginia], and 2008 to 2009, *n* = 5 [48]). All clinical samples were collected as a part of previous studies that were reviewed and approved by Institutional Biosafety and Human Investigation Committees. When required, genomic DNA was extracted using a QIAamp DNA minikit (Qiagen, Valencia, CA, USA) according to the manufacturer's instructions and eluted in 100 μl of elution buffer.

### Confirmation by Sanger sequencing.

Mutations present in the control P. falciparum lines and a subset of clinical samples (42/87) were confirmed by Sanger sequencing. First, the resistance-associated genes and genotyping SNPs were PCR amplified using primers described in Table S5 in the supplemental material (*pfCRT* [chloroquine], *pfCYTB* [atovaquone], *pfDHFR* [pyrimethamine], *pfDHPS* [sulfadoxine], *pfMDR1* [multidrug resistant], *pfKelch13* [artemisinin], *Pf*_01_000130573, *Pf*_01_000539044, *Pf*_02_000842803, *Pf*_06_000145472, *Pf*_06_000937750, and *Pf*_07_000277104). Each 25-μl PCR mixture contained 12.5 μl of HotStarTaq master mix (Qiagen, Valencia, CA, USA), 0.45 μl of the forward and reverse 50 μM primers (final concentration of 0.9 μM), 6.6 μl of nuclease-free water, and 5 μl of genomic DNA (500 pg total for DNA derived from laboratory parasites). PCR was performed on a CFX96 instrument (Bio-Rad, Hercules, CA, USA) and included an initial denaturation step at 95°C for 15 min, followed by 40 cycles of denaturation at 95°C for 30 s, annealing at 59°C for 30 s, and extension at 72°C for 30 s, with a final extension step at 72°C for 10 min. Next, PCR products were analyzed on 2% agarose gels, and verified PCR products were purified using a MinElute 96 UF PCR purification kit (Qiagen, Valencia, CA, USA) according to the manufacturer's protocol. Finally, purified PCR products were measured spectrophotometrically, diluted with nuclease-free water, mixed with primers, and then submitted to GeneWiz (Genewiz, Inc., South Plainfield, NJ, USA) for DNA sequencing. Sensitivity (the ability to correctly classify a mutant allele) and specificity (the ability to correctly classify a wild-type allele) were determined using a two-by-two table where the gold standard is Sanger sequencing. Accuracy (the ability to correctly classify both alleles) is an average of sensitivity and specificity values.

### Drug resistance loci and mutant allele selection.

We selected the targets for the initial malaria TAC design by (i) identifying previously reported associations with antimalarial resistance and (ii) prioritizing candidates based on global minor allele frequencies (MAF) reported in the MalariaGEN database (39). For *pfCRT*, *pfDHFR*, *pfDHPS*, and *pfMDR1*, most of the selected targets displayed a global MAF of >0.1 (5 of 26 total alleles) (Table S6). Since *pfKelch13* mutations have only recently arisen predominantly in Southeast Asia, global frequencies of all these are low (<0.1). We therefore included *pfKelch13* alleles that have been confirmed to confer artemisinin resistance (Y493H, R539T, I543T, and C580Y) (14, 49) as well as one allele that is associated with clinically delayed parasite clearance (F446I) (10). Additionally, in order to provide a complete picture of variation across this gene, we included mutations that have been observed in Asia and Africa but at the time of selection had an unknown association with resistance (N458Y, R561H, P574L, and A578S) (50–52). Since data were not available for *pfCYTB* mutations in the MalariaGen database, we directly selected atovaquone resistance-associated alleles from the literature (18–20).

### Assay development in 384-well plate format.

Primers and TaqMan probes for the malaria TAC assays were either designed using Primer Express3 (44 of 87 assays; Applied Biosystems, Life Technologies Corporation, Carlsbad, CA, USA) or adopted from published sources (43 of 87 assays [23, 36, 53–59]) (Table S7). Wild-type probes were designed based on the P. falciparum 3D7 sequences of the *pfCRT* (GenBank accession number NC_004328.2:458600-461695, gene ID 2655199), *pfCYTB* (AF069605.1), *pfDHFR* (NC_004318.1:755069-756895, gene ID 9221804), *pfDHPS* (NC_004329.2:549321-551737, gene ID 2655294), *pfMDR1* (NC_004326.1:957885-962144, gene ID 813045), and *pfKelch13* (NC_004331.2:1724848-1727028, gene ID 814205) genes. Six primer sets/12 probes for genotyping SNPs, 5 primer sets/probes for species identification, and the human GAPDH internal control (hGAPDH) were described in previous publications (36, 57–59).

Optimization of conditions and probe specificity testing were performed using the 384-well plate on the ViiA7 platform (Applied Biosystems, Life Technologies Corporation, Carlsbad, CA, USA). Each assay was amplified in duplex (Fig. 1 shows pairings), except for assays involved in species identification and the hGAPDH control. Primer/probe sets (0.09 μl of each forward and reverse primer, 0.025 μl of each probe of 50 μM stock, final concentrations of 0.9 μM and 0.25 μM, respectively) were assayed in a 5-μl PCR mixture containing 2.5 μl of 2× TaqMan universal master mix II (Applied Biosystems, Life Technologies Corporation, Carlsbad, CA, USA), 1.27 μl of nuclease-free water, and 1 μl (1 ng/μl) of genomic DNA. Cycling conditions included an initial denaturation at 95°C for 10 min, followed by 40 cycles of denaturation at 95°C for 15 s and annealing/extension at 59°C for 1 min. DNA sources included either P. falciparum 3D7 DNA as a wild-type control, 10 sequence-confirmed mutant P. falciparum lines (including Dd2, V1/S, 7G8, TMC90C6B, K1, HB3, PAIL, PUR, MON, and BAT), P. vivax, P. knowlesi, synthetic mutant control plasmids and species identification plasmids (P. malariae and P. ovale), a mixture of parasite-human DNA to test the performance of assays in the presence of human DNA (see below for ratios), or nuclease-free water as a nontemplate control.

Assay amplification efficiency and limits of detection were first performed on the 384-well plate format and subsequently on the array card format. To do so, DNA from individual parasite lines or plasmid controls was 10-fold serially diluted (plasmid control range, 10^5^ to 1 copy/μl; parasite DNA range, 1 ng to 10 fg or the equivalent of 4.03 × 10^4^ to 0.403 copies/μl). For 384-well plate assays, 1 μl of diluted samples was tested in each 5-μl reaction mixture in triplicate. Since the volume of DNA used in the array card is 5-fold lower (0.2 μl/reaction mixture), dilutions for malaria TAC testing were prepared as 5-fold more concentrated to ensure equivalence on both formats. The copy number of plasmid controls and copy number equivalents for parasite DNA were calculated using an available online tool (University of Rhode Island Genomics and Sequencing Center calculator [http://cels.uri.edu/gsc/cndna.html]) by inputting the amount of DNA in nanograms and the length in base pairs (23 Mb was used for parasite DNA). The formula is a follows: number of copies = (amount of DNA × 6.022 × 10^23^)/(length of template × 1 × 10^9^ × 650). To determine whether human DNA in the clinical samples impacted the performance of our assays, five randomly chosen assays were tested in duplicate in the presence and absence of human DNA (*pfCRT* 72C/72Stct, *pfDHFR* 164I/164L, *pfDHPS* 540K/540E, *pfMDR1* 86N/86Y, and P. falciparum). This was performed at multiple ratios of parasite/human DNA; human DNA was fixed at 10 ng (2,860 genome copies), and parasite DNA varied from 1 ng to 1 pg (40,300 to 40.3 genome copies), yielding the following ratios: 14:1, 1.4:1, 0.14:1, and 0.014:1.

### Evaluation of the malaria TAC.

Primer and TaqMan probe oligonucleotides were custom ordered, synthesized, and spotted into the microfluidic card by Applied Biosystems (Life Technologies Corporation, Carlsbad, CA, USA) as laid out in Fig. 1. Twenty to 50 μl of input DNA (at 1 ng/μl for culture parasite-derived DNA) was mixed with 50 μl of 2× TaqMan universal master mix II (Applied Biosystems, Life Technologies Corporation, Carlsbad, CA, USA) and 0 to 30 μl of nuclease-free water to yield a 100-μl final volume. This mixture was loaded into each port of the card; each card included a port for seven clinical samples and one synthetic positive-control plasmid (8 ports total). The loaded card was centrifuged twice at 1,200 rpm for 1 min and then sealed. The loading ports were excised, and the full card was inserted into a ViiA7 instrument (Life Technologies Corporation, Carlsbad, CA, USA) and run under the same cycling conditions as described above for 45 cycles. The results were automatically analyzed by ViiA7 software; the baseline and threshold were adjusted in cases where minor cross-reactivity occurred.

### Statistical analysis.

Means or medians were compared using Student's *t* test or a Mann-Whitney test. Data are shown as means ± standard deviations unless otherwise stated. A standard curve of hGAPDH was generated with known DNA concentrations and plotted against the *C_T_* value to yield the following equation: copy number per reaction product = 10^(*CT* − 37.65)/−3.6425^.

## Supplementary Material

Supplemental material
