# Supplementary material for: The Malaria TaqMan Array Card Includes 87 Assays for Plasmodium falciparum Drug Resistance, Identification of Species, and Genotyping in a Single Reaction
Source: Antimicrob Agents Chemother. 2017 Apr 24;61(5):e00110-17. doi: 10.1128/AAC.00110-17 (PMC5404514; doi:10.1128/AAC.00110-17)
Supplement: Supplemental material [file supp_61_5_e00110-17__index.html]

The Malaria TaqMan Array Card Includes 87 Assays for Plasmodium falciparum Drug Resistance, Identification of Species, and Genotyping in a Single Reaction — Supplemental material 

# The Malaria TaqMan Array Card Includes 87 Assays for Plasmodium falciparum Drug Resistance, Identification of Species, and Genotyping in a Single Reaction

## Supplemental material

- Supplemental file 1 -

  Tables S1-S7, Fig. S1-S4

  PDF, 2.0M
