# Supplementary material for: The Malaria TaqMan Array Card Includes 87 Assays for Plasmodium falciparum Drug Resistance, Identification of Species, and Genotyping in a Single Reaction
Source: Antimicrob Agents Chemother. 2017 Apr 24;61(5):e00110-17. doi: 10.1128/AAC.00110-17 (PMC5404514; doi:10.1128/AAC.00110-17)
Supplement: Supplemental material [file AAC.00110-17_zac005176164s1.pdf]

Table S1. PCR performance of each assay on the 384 well plate format

Assays	Linearity; R^2 (efficiency; %)		LOD, copies / reaction [a]		Assays	Linearity; R^2 (efficiency; %)		LOD, (copies / reaction)[a]	
Target1 / Target2	Target1	Target2	Target1	Target2	Target1 / Target2	Target1	Target2	Target1	Target2
pfCRT 72C / 72S(tct)	0.971 (95.0)	0.990 (81.9)	403	100	*pfMDR1* 1042N / 1042D	0.996 (95.6)	0.984 (86.8)	40.3	40.3
pfCRT 72S(agt) / 73-76 VMNK	0.982 (87.1)	0.978 (90.5)	403	403	*pfMDR1* 1246D / 1246Y	0.995 (89.6)	0.982 (88.0)	40.3	40.3
pfCRT 73-76 VMNT / VMET	0.992 (92.4)	0.998 (86.7)	100	100	*pfKelch13* 446F / 446I	0.965 (92.7)	0.998 (87.6)	4.03	100
pfCRT 73-76 VIET / VIDT	0.992 (90.5)	0.971 (83.1)	40.3	100	*pfKelch13* 458N / 458Y	0.998 (89.0)	0.995 (87.8)	40.3	100
pfCRT 97H / 97Q	0.987 (83.7)	0.998 (81.8)	40.3	100	*pfKelch13* 493Y / 493H	0.994 (85.6)	0.998 (87.1)	40.3	100
pfCRT 326N / 326S	0.981 (91.5)	0.993 (91.7)	40.3	40.3	*pfKelch13* 539R / 539T	0.995 (91.4)	0.995 (91.8)	40.3	40.3
pfCRT 326D / 356I	0.986 (99.1)	0.997 (93.7)	40.3	40.3	*pfKelch13* 543I / 543T	0.991 (98.5)	0.993 (90.3)	40.3	40.3
pfCRT 356T / 356L	0.998 (88.7)	0.997 (89.9)	4.03	4.03	*pfKelch13* 561R / 561H	0.972 (96.4)	0.996 (88.0)	4.03	100
pfCYTB 258I / 258M	0.990 (84.9)	0.992 (87.7)	4.03	10	*pfKelch13* 574P / 574L	0.995 (84.4)	0.995 (90.5)	4.03	10
pfCYTB 268Y / 286S	0.999 (88.8)	0.999 (90.2)	4.03	4.03	*pfKelch13* 578A / 578S	0.999 (90.5)	0.991 (80.1)	40.3	100
pfCYTB 268C / 268N	0.998 (89.4)	0.998 (84.1)	100	100	*pfKelch13* 580C / 580Y	0.981 (93.5)	0.999 (93.3)	40.3	40.3
pfCYTB 272K / 272R	1.000 (86.1)	0.997 (87.8)	4.03	10	*Pf*_01_000130573 C /T	0.984 (81.9)	0.999 (96.1)	40.3	40.3
pfDHFR 51N / 51I	0.982 (88.0)	0.997 (86.9)	40.3	40.3	*Pf*_01_000539044 A /G	0.994 (89.4)	0.992 (84.3)	40.3	40.3
pfDHFR 59C / 59R	0.995 (86.7)	0.991 (89.7)	40.3	40.3	*Pf*_02_000842803 T /C	0.990 (89.9)	0.998 (91.3)	40.3	40.3
pfDHFR 108S / 108N	0.986 (90.3)	0.993 (84.1)	40.3	40.3	*Pf*_06_000145472 G /C	0.997 (92.2)	0.984 (104.9)	40.3	40.3
pfDHFR 164I / 164L	0.996 (84.4)	0.995 (97.7)	403	403	*Pf*_06_000937750 A /G	0.992 (102.3)	0.995 (93.1)	40.3	40.3
pfDHPS 436-437SG / 436AG	0.990 (91.0)	0.999 (90.1)	40.3	4.03	*Pf*_07_000277104 A /G	0.948 (92.3)	0.993 (89.1)	40.3	40.3
pfDHPS 436-437SA/ 613T	0.994 (86.3)	0.997 (89.6)	40.3	40.3	*P. falciparum*	0.998 (90.1)	NA	40.3	NA
pfDHPS 540K / 540E	0.992 (81.7)	0.993 (89.9)	403	403	*P. vivax*	0.967 (128.8)	NA	421	NA
pfDHPS 581A /581G	0.988 (83.5)	0.975 (94.9)	40.3	40.3	*P. knowlesi*	0.965 (86.1)	NA	38.6	NA
pfDHPS 613A / 613S	0.999 (91.7)	0.976 (86.7)	4.03	4.03	*P. malariae*	0.993 (80.4)	NA	100	NA
pfMDR1 86N / 86Y	0.994 (85.5)	0.988 (87.9)	40.3	40.3	*P. ovale*	0.994 (81.4)	NA	10	NA
pfMDR1 184Y / 184F	0.987(89.0)	0.990 (86.2)	40.3	40.3	Human *GAPDH*	0.979 (101.6)	NA	2.8	NA
pfMDR1 1034S / 1034C	0.998 (83.8)	0.991 (92.8)	40.3	4.03					
Average ± SD					0.990 ± 0.01 (90% ± 6.4)				

LOD, limit of detection; NA, not applicable

[a] copy number of plasmid (10 - 100) or copy equivalent number of genomic DNA (4.3 - 403; calculated from 23 Mb genome size), *P. vivax* calculated from 22 Mb genome size, *P. knowlesi* calculated from 24 Mb genome size, human DNA calculated from 3,234.83 Mb genome size

Table S2. PCR performance of each assay on the TaqMan Array Card format

Assays	Linearity; R^2 (efficiency; %)		LOD, copies / reaction [a]		Assays	Linearity; R^2 (efficiency; %)		LOD, copies / reaction [a]	
Target1 / Target2	Target1	Target2	Target1	Target2	Target1 / Target2	Target1	Target2	Target1	Target2
pfCRT 72C / 72S(tct)	0.964 (83.4)	0.979 (102.6)	403	100	*pfMDR1* 1042N / 1042D	0.999 (85.6)	0.997 (108.5)	40.3	100
pfCRT 72S(agt) / 73-76 VMNK	0.988 (84.4)	0.995 (82.7)	100	403	*pfMDR1* 1246D / 1246Y	0.946 (98.7)	1.000 (102.2)	40.3	100
pfCRT 73-76 VMNT / VMET	0.998 (91.1)	0.998 (91.6)	100	100	*pfKelch13* 446F / 446I	0.999 (91.6)	0.998 (99.1)	4.03	100
pfCRT 73-76 VIET / VIDT	0.993 (96.3)	0.996 (91.1)	100	100	*pfKelch13* 458N / 458Y	0.991 (95.8)	0.982 (99.0)	40.3	100
pfCRT 97H / 97Q	0.999 (80.8)	0.991 (84.3)	40.3	100	*pfKelch13* 493Y / 493H	0.998 (81.9)	0.997 (94.9)	40.3	100
pfCRT 326N / 326S	0.999 (81.3)	0.996 (119.7)	40.3	100	*pfKelch13* 539R / 539T	0.995 (82.1)	0.987 (82.5)	40.3	100
pfCRT 326D / 356I	0.979 (90.2)	0.999 (86.3)	100	40.3	*pfKelch13* 543I / 543T	0.995 (92.9)	0.997 (94.2)	40.3	100
pfCRT 356T / 356L	0.999 (92.1)	0.999 (91.9)	10	10	*pfKelch13* 561R / 561H	0.998 (88.6)	0.987 (98.6)	4.03	100
pfCYTB 258I / 258M	0.999 (91.9)	0.997 (99.8)	4.03	10	*pfKelch13* 574P / 574L	0.997 (91.8)	0.999 (93.9)	4.03	10
pfCYTB 268Y / 286S	0.996 (92.0)	0.996 (96.1)	4.03	10	*pfKelch13* 578A / 578S	0.998 (82.5)	0.990 (86.0)	40.3	100
pfCYTB 268C / 268N	0.990 (107.5)	0.981 (119.2)	100	100	*pfKelch13* 580C / 580Y	0.998 (84.3)	0.998 (90.1)	40.3	100
pfCYTB 272K / 272R	0.993 (95.2)	0.999 (91.1)	4.03	10	*Pf*_01_000130573 C /T	0.999 (81.2)	0.999 (88.9)	40.3	100
pfDHFR 51N / 51I	0.999 (83.1)	0.999 (88.3)	40.3	100	*Pf*_01_000539044 A /G	0.999 (83.9)	0.999 (77.6)	100	40.3
pfDHFR 59C / 59R	0.997 (88.1)	0.999 (94.6)	40.3	100	*Pf*_02_000842803 T /C	0.997 (88.6)	0.999 (84.1)	100	40.3
pfDHFR 108S / 108N	0.999 (76.2)	0.995 (80.1)	40.3	100	*Pf*_06_000145472 G /C	0.999 (93.7)	0.999 (89.5)	100	40.3
pfDHFR 164I / 164L	0.998 (89.0)	0.995 (100.1)	403	100	*Pf*_06_000937750 A /G	0.992 (93.6)	0.993 (94.5)	100	40.3
pfDHPS 436-437SG / 436AG	0.999 (83.5)	0.998 (93.4)	40.3	100	*Pf*_07_000277104 A /G	0.998 (95.7)	0.998 (87.3)	100	40.3
pfDHPS 436-437SA/ 613T	0.999 (88.6)	0.998 (82.3)	100	100	*P. falciparum*	0.999 (88.8)	NA	40.3	NA
pfDHPS 540K / 540E	0.998 (93.4)	0.998 (90.1)	403	100	*P. vivax*	0.998 (98.6)	NA	100	NA
pfDHPS 581A /581G	0.995 (87.7)	0.991 (95.2)	40.3	100	*P. knowlesi*	0.996 (101.1)	NA	10	NA
pfDHPS 613A / 613S	0.999 (91.0)	0.996 (105.2)	4.03	100	*P. malariae*	0.999 (90.1)	NA	100	NA
pfMDR1 86N / 86Y	0.989 (85.3)	0.984 (93.4)	40.3	100	*P. ovale*	0.998 (94.6)	NA	10	NA
pfMDR1 184Y / 184F	0.947 (91.7)	0.996 (90.1)	40.3	100	Human *GAPDH*	0.998 (88.2)	NA	10	NA
pfMDR1 1034S / 1034C	0.999 (91.2)	0.991 (104.0)	40.3	100					
Average ± SD					0.994 ± 0.01 (92% ± 7.9)				

LOD; limit of detection, NA; not applicable

[a] copy number of plasmid (10 - 100) or copy equivalent number of genomic DNA (4.3 - 403; calculated from 23 Mb genome size)

Table S3. Performance of malaria-TAC to detect drug resistance on clinical samples from each origin

Targets	Thailand (n=32)			China (n=21)			Malawi (n=28)			Uganda (n=6)		
	Detectable		Ct (Ẋ ± SD)	Detectable		Ct (Ẋ ± SD)	Detectable		Ct (Ẋ ± SD)	Detectable		Ct (Ẋ ± SD)
	N	%		N	%		N	%		N	%	
Human *GAPDH*	28	87	32.1 ± 1.9	21	100	27.5 ± 1.8	25	89	33.5 ± 1.0	6	100	24.8 ± 1.5
P. vivax	1	100	29.2	-	-	-	-	-	-	-	-	-
P. knowlesi	-	-	-	-	-	-	-	-	-	-	-	-
P. ovale	-	-	-	-	-	-	-	-	-	1	100	29.5
P. falciparum	30	97	32.1 ± 1.8	21	100	22.6 ± 3.4	25	89	32.9 ± 3.5	5	100	23.6 ± 3.6
pfCRT 72-76	27	87	35.9 ± 2.5	14	67	34.4 ± 4.3	16	57	35.9 ±3.5	5	100	26.1 ± 4.8
pfCRT 97	23	74	39.7 ± 2.6	8	38	37.0 ± 3.2	20	71	37.5 ±4.4	5	100	28.9 ± 4.8
pfCRT 326	31	100	34.6 ± 2.4	21	100	27.6 ± 5.0	23	82	33.9 ±3.5	5	100	25.2 ± 3.6
pfCRT 356	31	100	33.8 ± 2.3	21	100	26.5 ± 5.5	23	82	34.8 ±2.9	5	100	26.4 ± 4.1
pfCYTB 258	31	100	25.5 ± 1.9	21	100	22.4 ± 5.7	28	100	27.9 ±3.7	5	100	20.8 ± 5.4
pfCYTB 268	31	100	26.1 ± 2.1	18	86	26.0 ± 4.5	27	96	28.7 ± 3.8	5	100	20.5 ± 4.8
pfCYTB 272	31	100	27.1 ± 2.2	20	95	26.9 ± 5.7	27	96	30.0 ±3.9	5	100	22.3 ± 5.4
pfDHFR 51	30	97	37.5 ± 2.3	14	67	33.8 ± 4.4	23	82	36.2 ±3.3	5	100	27.7 ± 5.0
pfDHFR 59	28	90	35.1 ± 2.6	21	100	32.7 ± 6.5	22	79	34.1 ±2.9	5	100	26.0± 4.4
pfDHFR 108	30	97	37.5 ± 2.7	14	67	35.2 ± 5.0	21	75	36.2 ± 3.2	5	100	27.9 ± 4.7
pfDHFR 164	30	97	35.7 ± 2.6	8	38	32.7 ± 6.6	23	82	33.8 ± 3.8	5	100	25.5 ±4.5
pfDHPS 436-437	30	97	33.2 ± 1.8	21	100	26.1 ± 3.5	23	82	33.1 ± 3.3	5	100	25.9 ± 4.5
pfDHPS 540	26	84	38.3 ± 3.0	8	38	35.0 ± 4.8	22	79	35.8 ±3.1	5	100	27.7 ± 5.0
pfDHPS 581	29	94	34.3 ± 2.2	19	90	30.7 ± 7.7	20	71	33.2 ± 3.3	5	100	26.3 ± 5.0
pfDHPS 613	30	97	33.2 ± 1.8	21	100	24.4 ± 3.6	21	75	32.6 ±2.7	5	100	26.0 ± 5.1
pfMDR1 86	28	90	33.7 ± 2.4	21	100	27.5 ± 4.1	24	86	34.2 ±3.1	5	100	27.4 ± 4.6
pfMDR1 184	30	97	34.1 ± 3.7	19	90	32.8 ± 5.5	20	71	34.3 ±3.2	5	100	27.5 ±5.8
pfMDR1 1034	30	97	34.1 ± 2.3	18	86	31.6 ± 7.4	21	75	33.5 ± 3.7	5	100	24.7 ±4.6

pfMDR1 1042	30	97	34.6 ± 3.1	17	81	33.2 ± 4.6	22	79	33.7 ± 4.1	5	100	25.2 ±4.4
pfMDR1 1246	29	94	35.9 ± 2.8	19	90	33.1 ± 6.1	22	79	35.2 ± 4.0	5	100	30.9 ±6.3
pfKelch13 446	29	94	33.0 ± 2.4	21	100	29.5 ± 6.0	21	75	32.7 ±3.6	5	100	23.9 ± 4.9
pfKelch13 458	31	100	35.8 ± 2.9	21	100	33.2 ± 6.1	20	71	35.1 ±2.7	5	100	27.0 ± 4.7
pfKelch13 493	30	97	33.8 ± 2.6	21	100	28.5 ± 5.0	22	79	33.5 ± 3.2	5	100	25.6 ± 4.8
pfKelch13 539	30	97	34.8 ± 2.0	20	95	31.9 ± 6.1	22	79	34.5 ± 2.8	5	100	27.1 ± 4.9
pfKelch13 543	30	97	31.6 ± 1.7	21	100	25.4 ± 4.3	18	64	31.7 ±3.9	5	100	24.4 ±5.1
pfKelch13 561	29	94	31.5 ± 1.9	21	100	25.9 ± 5.6	24	86	31.9 ±2.9	5	100	23.7 ± 4.8
pfKelch13 574	31	100	32.2 ± 2.0	21	100	25.5 ± 5.0	21	75	31.1 ±2.9	5	100	25.1 ± 5.4
pfKelch13 578	29	94	32.7 ± 1.7	21	100	25.5 ± 4.3	21	75	32.9 ±3.5	5	100	25.7 ±4.8
pfKelch13 580	30	97	34.6 ± 2.0	21	100	26.1 ± 4.4	22	79	34.3 ± 3.5	5	100	27.3 ±5.7
*Pf*_01_000130573	31	100	34.3 ± 1.9	21	100	25.3 ± 4.1	24	86	34.2 ± 3.0	5	100	26.5 ±5.5
*Pf*_01_000539044	28	90	37.1 ± 2.7	21	100	28.8 ± 4.8	19	68	36.6 ± 3.0	5	100	29.4 ±6.0
*Pf*_02_000842803	30	97	35.1 ± 2.3	21	100	25.6 ± 3.6	18	64	34.8 ±3.0	5	100	27.7 ± 4.5
*Pf*_06_000145472	29	94	32.8 ± 2.1	21	100	25.2 ± 4.7	21	75	32.3 ±2.5	5	100	25.4 ± 5.0
*Pf*_06_000937750	30	97	35.4 ± 2.7	21	100	30.0 ± 4.6	20	71	33.9 ±2.9	5	100	27.2 ±4.4
*Pf*_07_000277104	29	94	34.1 ± 1.9	21	100	28.5 ± 4.9	20	71	34.0 ± 3.5	5	100	28.2 ± 5.8
Ẋ ± SD		95 ± 5.3			90 ± 18.4			78 ± 9.0			100	

Ct; cycle threshold, Ẋ; mean, SD; standard deviation

Table S4. Comparison of mutant allele patterns by gene and origin

Gene	Origin	N[a]	Allele[b] 72-76 (CVMNK)	97 (H)	326 (N)	356 (I)
pfCRT	Thailand	23	CVIET	H	S	T
	China	8	CVIET	H	S	T
	Malawi	15	CVMNK	H	N	I
	Uganda	5	CVIET	H	N	I
	Total	51				
			51 (N)	59 (C)	108 (S)	164 (I)
pfDHFR	Thailand	21	I	R	N	L
		1	I	R	N	I/L
		3	N	R	N	I
		1	N	R	N	L
		1	I	R	N	I
	China	4	I	R	N	L
		3	N	R	N	L
	Malawi	18	I	R	N	I

Gene	Origin	N[a]	Allele[b] 436 (S)	437 (G)	540 (K)	581 (A)	613 (A)
pfDHPS	Thailand	22	S	G	E	G	A
		1	A	G	E	A	A
		1	S/A	G	E	A/G	A
	China	6	A	G	E	A	A
		2	S	G	E	G	A
	Malawi	11	S	G	E	A	A
		6	S	G	E	G	A
	Uganda	5	S	G	E	A	A
	Total	54					
			86 (N)	184 (Y)	1034 (S)	1042 (N)	1246 (D)
pfMDR1	Thailand	16	N	Y	S	N	D
		8	N	F	S	N	D
		1	N	Y/F	S	N	D
		1	N	F	S	D	D
	China	8	N	Y	S	N	D
		7	N	F	S	N	D
	Malawi	3	N	Y	S	N	D
		12	N	F	S	N	D

Gene	Origin	N[a]	Allele[bc] 446 (F)	458 (N)	493 (Y)	539 (R)	543 (I)	561 (R)	574 (P)	578 (A)	580 (C)
pfKelch13	Thailand	24	F	N	Y	R	I	R	P	A	C
		1	F	N	Y	R	I	R	L	A	C
		1	I	N	Y	R	I	R	P	A	C
	China	3	F	N	Y	R	I	R	P	A	C
		14	I	N	Y	R	I	R	P	A	C
		2	F	N	Y	R	I	R	L	A	C
		1	F	N	Y	R	I	H	P	A	C
	Malawi	13	F	N	Y	R	I	R	P	A	C
		1	F	N	Y	R	I	R	P	S	C
	Uganda	5	F	N	Y	R	I	R	P	A	C
	Total	65									
			258 (I)	268 (Y)	272 (K)						
pfCYTB	Thailand	31	I	Y	K						
	China	18	I	Y	K						
	Malawi	27	I	Y	K						
	Uganda	5	I	Y	K						
	Total	81									

Uganda	3	I	R	N	I		1	N	Y/F	S	N	D
	1	I	C/R	N	I	Uganda	2	Y	Y	S	N	Y
	1	I	C	N	I		1	Y	F	S	N	D
Total	57						1	Y	Y/F	S	N	D/Y
							1	N/Y	Y/F	S	N	D/Y
						Total	62					

[a] incomplete data for all codons of each gene was excluded

[b] amino acid in parentheses are wild-type, A/B indicated hetero-resistance, One sample that detected F446I was not shown since there was incomplete data for all codons.

[c] Y493H, R539T, I543T, and C580Y are confer to artemisinin resistance (1, 2), F446I is associated to the delayed parasite clearance (3), Shaded N458Y, R561H, P574L, and A578A have an unknown contribution to artemisinin resistance (4-6)

Table S5. Sequencing primers

Gene	Sequences (5’-3’)	Product size (bp)
pfCRT 72-97	For TCTTGGTAAATGTGCTCATGTGTTT	251
	Rev TTTATCTTACTTTTGAATTTCCCTTTT	
pfCRT 326-356	For TTTTTAGAAAACCTTCGCATTGTT	308
	Rev TACGGCTAAGAATTTAAAGTAATAAGC	
pfCYTB 258-272	For CAGTAATTTGGATATGTGGAGGA	562
	Rev TGTGGTAATTGACATCCAATCC	
pfDHFR 51-164	For AAGCAAAAATGAGGGGAAAAA	576
	Rev ACATCGCTAACAGAAATAATTTGA	
pfDHPS 436-540	For CCTAAACGTGCTGTTCAAAGAA	476
	Rev CGAGGTATTCCATTTAATACAAGAAAA	
pfDHPS 581-613	For TTCTTGTATTAAATGGAATACCTCGTT	229
	Rev TTTTCATTTTGTTGTTCATCATGT	
pfMDR1 86-184	For TGAACAAAAAGAGTACCGCTGA	549
	Rev AAATTAACGGAAAAACGCAAG	
pfMDR1 1034-1042	For CAAGCGGAGTTTTTGCATTT	595
	Rev CCTGTTTCTCCAACGATTGC	
pfMDR1 1246	For GCAATCGTTGGAGAAACAGG	562
	Rev TGGAATCAAGTGATGATGTTGC	
pfKelch13 446-580	For TCCGTTAACTATACCCATACCAAAA	645
	Rev GCTGCTCCTGAACTTCTAGCTT	
*Pf*_01_000130573	For TCAATGGGTATCTCAGGTTTTT	562
	Rev CGGAAGAAGCTGTGATGAATG	
*Pf*_01_000539044	For TGATAAAACCAAGAAGTCCAAGAA	160
	Rev CAAATGGCATCCACTGAAAA	
*Pf*_02_000842803	For CACAATTCAATGGCATCAAGA	357

	Rev AGCAACTGCTTGGTATTCACA	
*Pf*_06_000145472	For CCAATCAACAAAAGCTGATGAA	221
	Rev TTTCCAAGGAAGGTAGAATATCAAA	
*Pf*_06_000937750	For TCATTTTTAGGAGGCATAAATCC	572
	Rev AAAAACACATTGACTATTCCTTCAA	
*Pf*_07_000277104	For AATTAATATTATGAATATGAGTACTCAGCATTATATCTT	143
	Rev ACGTCCATATGTTGAAGATGATTGCA	

For; forward, Rev; reverse

Table S6. Minor allele frequencies (MAF) reported in MalariaGEN database (7)

Gene	Mutation	Minor allele frequencies								
		WAF	CAF	EAF	SAS	WSEA	ESEA	OCE	SAM	Global
pfCRT	C72Stct	0.0	0.0	0.0	0.0	0.0	0.0	0.0	0.259	0.032
	C72Sagt	0.0	0.001	0.0	0.0	0.0	0.0	0.031	0.0	0.121
	M74I	0.247	0.339	0.055	0.300	0.005	0.089	0.0	0.0	0.406
	N75E	NA	NA	NA	NA	NA	NA	NA	NA	NA
	N75D	0.239	0.369	0.055	0.353	0.005	0.092	0.0	0.407	0.476
	K76T	0.243	0.343	0.057	0.300	0.008	0.089	0.030	0	0.348
	H97Q	0.0	0.0	0.0	0.0	0.0	0.0	0.0	0.407	0.074
	N326S	0.015	0.0	0.0	0.282	0.002	0.438	0.001	0.0	0.232
	N326D	0.0	0.0	0.0	0.0	0.0	0.0	0.030	0.407	0.172
	I356T	0.173	0.264	0.0	0.162	0.007	0.434	0.0	0.0	0.354
	I356L	0.0	0.0	0.0	0.0	0.0	0.0	0.029	0.407	0.172
pfDHFR	N51I	0.305	0.025	0.049	0.469	0.084	0.102	0.0	0.372	0.341
	C59R	0.227	0.228	0.058	0.023	0.0	0.008	0.009	0.0	0.194
	S108N	0.205	0.006	0.017	0.003	0.0	0.005	0.007	0.037	0.035
	I164L	0.0	0.0	0.0	0.366	0.152	0.349	0.0	0.111	0.209
pfDHPS	S436A	0.476	0.056	0.028	0.498	0.227	0.340	0.0	0.0	0.209
	G437A	0.231	0.053	0.073	0.127	0.0	0.148	0.454	0.333	0.231
	K540E	0.011	0.084	0.110	0.211	0.100	0.332	0.369	0.148	0.440
	A581G	0.023	0.029	0.059	0.192	0.250	0.316	0.0	0.259	0.204
	A613S	0.108	0.0	0.0	0.0	0.0	0.034	0.0	0.0	0.018
pfMDR1	N86Y	0.240	0.445	0.187	0.195	0.005	0.004	0.293	0.0	0.223
	Y184F	0.339	0.359	0.427	0.183	0.207	0.455	0.121	0.0	0.427
	S1034C	0.0	0.0	0.0	0.0	0.002	0.0	0.0	0.296	0.037
	N1042D	0.0	0.0	0.0	0.0	0.032	0.010	0.109	0.037	0.139

	D1246Y	0.037	0.092	0.117	0.0	0.002	0.0	0.0	0.407	0.082
pfKelch13	F446I	0.0	0.0	0.0	0.0	0.011	0.0	0.0	0.0	0.001
	N458Y	0.0	0.0	0.0	0.0	0.022	0.0	0.0	0.0	0.003
	Y493H	0.0	0.0	0.0	0.0	0.0	0.058	0.0	0.0	0.007
	R539T	0.0	0.0	0.0	0.0	0.002	0.048	0.0	0.0	0.006
	I543T	0.0	0.0	0.0	0.0	0.0	0.026	0.0	0.0	0.003
	R561H	0.0	0.0	0.0	0.0	0.043	0.0	0.0	0.0	0.005
	P574L	0.0	0.0	0.0	0.0	0.020	0.002	0.0	0.0	0.003
	A578S	0.005	0.005	0.005	0.0	0.0	0.0	0.0	0.0	0.002
	C580Y	0.0	0.0	0.0	0.0	0.115	0.296	0.0	0.0	0.051

WAF: West Africa, CAF: Central Africa, EAF: East Africa, SAS: South Asia, WSEA: West Southeast Asia, ESEA: East Southeast Asia, OCE: Oceana, SAM: South America

Table S7. Primers and probes sequences in Malaria-TAC

Gene	Primers/Probes	Sequences (5'-3')	Ref.
pfCRT	72-For	TGGTAAATGTGCTCATGTGTTT	(8, 9)
	72-Rev	AGTTTCGGATGTTACAAAACTATAGT	(8, 9)
	72C	FAM-5'-TYMATTACACATACACTTAAA-3'-MGB	This study
	72S(tct)	VIC-5'-TWTYMATTACAGATACACTTAAA-3'-MGB	This study
	72S(agt)	FAM-5'-TTTAAGTGTAAGTGTAATGAA-3'-MGB	This study
	73-76-For	TGGTAAATGTGCTCATGTGTTT	(8, 9)
	73-76-Rev	AGTTTCGGATGTTACAAAACTATAGT	(8, 9)
	73-76VMNK	VIC-5'-TGTAATGAATAAAATTTT-3'-MGB	This study
	73-76VMNT	FAM-5'-TGTAATGAATACAATTTT-3'-MGB	This study
	73-76VMET	VIC-5'-AWSTGTAATGGAAACAATT-3'-MGB	This study
	73-76VIET	FAM-5'-CAAAAATTGTTTCAATTACASWT-3'-MGB	This study
	73-76VIDT	VIC-5'-CAAAAATTGTATCAATTACACAT-3'-MGB	This study
	97-For	TGCTAAAAGAACTTTAAACAAAATTGG	This study
	97-Rev	TTTATCTTACTTTTGAATTTCCCTTTT	This study
	97H	FAM-5'-CCGAAACTCACAACTT-3'-MGB	This study
	97Q	VIC-5'-CGAAACTCARAACTTTAT-3'-MGB	This study
	326-For	TTTTTAGAAAACCTTCGCATTGTT	This study
	326-Rev	TCAACGTTTTTCATCCTTTTTATTCTT	This study
	326N	FAM-5'-TTCCTTCTTTAACATTTGTGATA-3'-MGB	This study
	326S	VIC-5'-TTCCTTCTTTAGCATTTGTGATA-3'-MGB	This study
	326D	FAM-5'-TTCCTTCTTTGACATTTGTGATA-3'-MGB	This study
	356-For	TCGACAAATTTTCTACCATGACA	This study
	356-Rev	TACGGCTAAGAATTTAAAGTAATAAGC	This study
	356I	VIC-5'-GTCCAGCAATAGC-3'-MGB	This study
	356T	FAM-5'-CCAGCAACAGCAATT-3'-MGB	This study
	356L	VIC-5'-GTCCAGCATTAGCAAT-3'-MGB	This study
pfCYTB	258-For	TTTATCACATCCTGATAATGCTATCG	This study
	258-Rev	CCAGCTGGTTTACTTGGAACA	This study
	258I	FAM-5'-CATCTCAAATTGTACCTGAAT-3'-MGB	This study
	258M	VIC-5'-CATCTCAAATGGTACCTGAA-3'-MGB	This study
	268-For	TTTATCACATCCTGATAATGCTATCG	This study
pfCYTB	268-Rev	CCAGCTGGTTTACTTGGAACA	This study
	286Y	FAM-5'-CCATTTTATGCAATGTT-3'-MGB	This study
	268S	VIC-5'-CTACCATTTTCTGCAATGT-3'-MGB	This study
	268C	FAM-5'-CTACCATTTTGTGCAATGT-3'-MGB	This study
	268N	VIC-5'-TCTACCATTTAATGCAATGT-3'-MGB	This study
	272-For	TTGTACCTGAATGGTACTTTCTACC	This study
	272-Rev	AGTTGTTAAACTTCTTTGTTCTGCT	This study
	272K	FAM-5'-CAATGTTAAAAACTGTTCCA-3'-MGB	This study
	272R	VIC-5'-CAATGTTAAGAACTGTTCC-3'-MGB	This study
pfDHFR	51-For	CACATTTAGAGGTCTAGGAAATAAAGG	This study
	51-Rev	CAATTTTTCATATTTTGATTCATTCAC	This study
	51N	FAM-5'-CATGGAAATGTAATTCCCTAGA-3'-MGB	This study
	51I	VIC-5'-CATGGAAATGTATTTCCCTAGA-3'-MGB	This study
	59-For	TGAGGTTTTTAATAACTACACATTTAGAGGTCT	(10)
	59-Rev	TATCATTTACATTATCCACAGTTTCTTTGTT	(10)
	59C	FAM-5'-ACTGCACAAAAATAT-3'-MGB	This study
	59R	VIC-5'-TAACTGCACGAAAAT-3'-MGB	This study
	108-For	TGGATAATGTAAATGATATGCCTAATTCTAA	(10)
	108-Rev	AATCTTCTTTTTTTAAGGTTCTAGACAATATAACA	(10)
	108S	FAM-5'-TTTCCCAGCTTGTTCT-3'-MGB	This study
	108N	VIC-5'-CTTTCCCAGTTTGTTCT-3'-MGB	This study
	164-For	GAAGATCTAATAGTTTTACTTGGGAA	This study
	164-Rev	TTTTCTAAAAATTCTTGATAAACAACG	This study
	164I	FAM-5'-ATGTTTTATTATAGGAGGTTC-3'-MGB	This study
	164L	VIC-5'-ATGTTTTATTTTAGGAGGTTC-3'-MGB	This study
pfDHPS	436-437-For	TGAAATGATAAATGAAGGTGCTAGTGT	(10)
	436-437-Rev	AATACAGGTACTACTAAATCTCTTTCACTAATTTTT	(10)
	436-437SG	FAM-5'-AGAATCCTCTGGTCCTT-3'-MGB	This study
	436-437AG	VIC-5'-AGAATCCGCTGGTCC-3'-MGB	This study
	436-437SA	FAM-5'-ATCCTCTGCTCCTTTT-3'-MGB	This study

For = forward, Rev = reverse, P = probe, Ref. = references, SNP = single nucleotide polymorphism, underline = codon or nucleotide position of mutation

Table S7. Primers and probes sequences in Malaria-TAC (continued)

Gene	Primers/Probes	Sequences (5’-3’)	Ref.	Gene	Primers/Probes	Sequences (5’-3’)	Ref.
pfDHPS	540-For	GCATAAAAGAGGAAATCCACATA	This study	*pfMDR1*	1246-For	CCAATCTGGATCTGCAGAAGATTA	This study
	540-Rev	CGAGGTATTCCATTTAATACAAGAAAA	This study		1246-Rev	ACATGGGTTCTTGACTAACTATTGAAA	This study
	540K	FAM-5'-CAATGGATAAACTAACAAA-3'-MGB	(10)		1246D	FAM-5'-TGATTATAACTTAAGAGATCTTAGAAA-3'-MGB	(11) Modified
	540E	VIC-5'-AATGGATGAACTAACAAA-3'-MGB	(10)		1246Y	VIC-5'-TTATAACTTAAGATATCTTAGAAAC-3'-MGB	(11) Modified
	581-For	CCTCGTTATAGGATACTATTTGATATTGGAT	(10)	*pfKelch13*	446-For	TGAAGCCTTGTTGAAAGAAGCA	This study
	581-Rev	TGGGCAATAAATCTTTTTCTTGAATA	(10)		446-Rev	TCCATCGAATTTAAATATTCTACACCA	This study
	581A	FAM-5'-TTCTTCGCAAATCC-3'-MGB	This study		446F	FAM-5'-TCCCATTAGTATTTTGTATAGGT-3'-MGB	This study
	581G	VIC-5'-GTTTCTTCCCAAATCCTA-3'-MGB	This study		446I	VIC-5'-TCCCATTAGTAATTTGTATAGGT-3'-MGB	This study
	613-For	ATATGATGAGTATCCACTTTTTATTGG	This study		458-For	TGAAGCCTTGTTGAAAGAAGCA	This study
	613-Rev	TTGTTGTTCATCATGTAATTTTTGTTG	This study		458-Rev	GGTGTACACATACGCCAGCA	This study
	613A	FAM-5'-CAATGGGCAATAAAT-3'-MGB	This study		458N	FAM-5'-CCATCGAATTTAAATATT-3'-MGB	This study
	613S	VIC-5'-CAATGGGAAATAAAT-3'-MGB	This study		458Y	VIC-5'-TCCATCGAATATAAATATT-3'-MGB	This study
	613T	VIC-5'-ATGCAATGGGTAATAA-3'-MGB	This study				
pfMDR1	86-For	TGTATGTGCTGTATTATCAGGAGGAAC	(12)		493-For	CAACAATGCTGGCGTATGTG	This study
	86-Rev	AATTGTACTAAACCTATAGATACTAATGATAATATTATAGG	(12)		493-Rev	AACGATCATACACCTCAGTTTCAA	This study
	86N	FAM-5'-ACCTAAATTCATGTTCTTT-3'-MGB	(12)		493Y	FAM-5'-ACCAAAAACGTATAAGAAA-3'-MGB	This study
	86Y	VIC-5'-CACCTAAATACATGTTCTT-3'-MGB	(12) Modified		493H	VIC-5'-CCAAAAACGTGTAAGAAA-3'-MGB	This study
	184-For	CAAGTGAGTTCAGGAATTGGTACG	This study		539-For	CCTAGAAGAAATAATTGTGGTGTTACG	This study
	184-Rev	CATAAATTAACGGAAAAACGCAAG	This study		539-Rev	GTGCCACCTCTACCCATGCT	This study
	184Y	FAM-5'-TTTTAGGTTTATATATTTGGTCATT-3'-MGB	This study		539R	FAM-5'-CAAATGGTAGAATTTATTG-3'-MGB	This study
	184F	VIC-5'-TTTTAGGTTTATTTATTTGGTCATT-3'-MGB	This study		539T	VIC-5'-CAAATGGTACAATTTATTG-3'-MGB	This study
	1034-For	AAAAAGAAGAATTATTGTAAATGCAGCTT	(12)		543-For	TTGTGGTGTTACGTCAAATGGT	This study
	1034-Rev	GGATCCAAACCAATAGGCAAAA	(12)		543-Rev	GTGCCACCTCTACCCATGCT	This study
	1034S	FAM-5'-TTTGACTGAATCCC-3'-MGB	This study		543I	FAM-5'-ATCCCCCAATACAATAA-3'-MGB	This study
	1034C	VIC-5'-TTTGACAGAATCCC-3'-MGB	This study		543T	VIC-5'-ATCCCCCAGTACAATAA-3'-MGB	This study
	1042-For	AAAAAGAAGAATTATTGTAAATGCAGCTT	(12)		561-For	TTGGGGGATATGATGGCTCT	This study
	1042-Rev	TTTCCAGCATAACTACCAGTAAATATAAAAG	(12)		561-Rev	GCTGATGATCTAGGGGTATTCAAA	This study
	1042N	FAM-5'-AGGCAAAACTATTAATAAATA-3'-MGB	This study		561R	FAM-5'-ATGATCATCGTATGAAAGC-3'-MGB	This study
	1042D	VIC-5'-AGGCAAAACTATCAATAAATA-3'-MGB	This study		561H	VIC-5'-ATGATCATCATATGAAAGC-3'-MGB	This study

For = forward, Rev = reverse, P = probe, Ref. = references, SNP = single nucleotide polymorphism, underline = codon or nucleotide position of mutation

Table S7. Primers and probes sequences in Malaria-TAC (continued)

Gene	Primers/Probes	Sequences (5'-3')	Ref.
pfKelch13	574-For	TGAAAGCATGGGTAGAGGTG	This study
	574-Rev	ATCTCTCACCATTAGTTCCACCAA	This study
	574P	FAM-5'-ATGATCTAGGGGTATTC-3'-MGB	This study
	574L	VIC-5'-TGATGATCTAAGGGTATTC-3'-MGB	This study
	578-For	TGAAAGCATGGGTAGAGGTG	This study
	578-Rev	ATCTCTCACCATTAGTTCCACCAA	This study
	578A	FAM-5'-AGATCATCAGCTATGTGT-3'-MGB	This study
	578S	VIC-5'-CTAGATCATCATCTATGTGTGTT-3'-MGB	This study
	580-For	GGTGGCACCTTTGAATACCC	This study
	580-Rev	ATCTCTCACCATTAGTTCCACCAAT	This study
	580C	FAM-5'-AGCTATGTGTGTTGCTT-3'-MGB	This study
	580Y	VIC-5'-CAGCTATGTATGTTGCTTT-3'-MGB	This study
SNP[a]	1-For	CTTCCTCTTACCCATATTTTCTTCTTTCCT	(13)
	1-Rev	TGTGAAAATGAGAAGAGAGAAATATGTGTTGA	(13)
	1C	FAM-5'-TCTTCATTATATGCATCTTTT-3'-MGB	(13)
	1T	VIC-5'-TCTTCATTATATGTATCTTTT-3'-MGB	(13) Modified
	2-For	CCAAGAAGTCCAAGAATAATAGTTACTATCACA	(13)
	2-Rev	TTCATGTGTGGCTGCTTTCCTAT	(13)
	2A	FAM-5'-CAACCGTTTCTTTAGCC-3'-MGB	(13)
	2G	VIC-5'-ACCGTTTCCTTAGCCTT-3'-MGB	(13) Modified
	3-For	ATTCCAAAACTATGTTTGCTGCTTTTCA	(13)
	3-Rev	AGAAATGCAGTGGTACTTGTTGCTA	(13)
	3T	FAM-5'-AGATTATTTGCATCATTACTT-3'-MGB	(13)

Gene	Primers/Probes	Sequences (5'-3')	Ref.
SNP[a]	7-For	CTAGCTCAGCTTCCAATTTGTCAAA	(13)
	7-Rev	GTCCACATCCATATACTGCTGTACAA	(13)
	7A	FAM-5'-TGTAGCAATACCCCCAAC-3'-MGB	(13) Modified
	7G	VIC-5'-TGTAGCAATGCCCCCAA-3'-MGB	(13) Modified
	8-For	AATTAATATTATGAATATGAGTACTCAGCATTATATCTT	(13)
	8-Rev	ACGTCCATATGTTGAAGATGATTGCA	(13)
	8A	FAM-5'-CCACCGAACTTATAT-3'-MGB	(13) Modified
	8G	VIC-5'-ACCGAACTCATATTTAT-3'-MGB	(13) Modified
18S	*P. falciparum*-For	CCACATCTAAGGAAGGCAGCAG	(14)
	P. falciparum-Rev	CCTCCAATTGTTACTCTGGGAAGG	(14)
	P. falciparum-P	FAM-5'-CCCACCATTCCAATTACAA-3'-MGB	(14)
AMA1	*P. vivax*-For	GGATGGGAACTGCGAAGAAA	This study
	P. vivax-Rev	TCCGTCATTTCTTCTTCATACTGAG	(14) Modified
	P. vivax-P	FAM-5'-ATCTGAGGCACTCGCT-3'-MGB	(14) Modified
Plasmepsin	*P. knowlesi*-For	CCTTTCCTTCCATTCTACGTAACC	This study
	P. knowlesi-Rev	GACGTCGAGAAGTGGGTTCA	This study
	P. knowlesi-P	FAM-5'-CAGCCAACAACACTTACA-3'-MGB	(14) Modified
Plasmepsin 4	*P. malariae*-For	GCCAACAATACATACACATTAGAACCA	(14) Modified
	P. malariae-Rev	CAATATCGACTGGTAGGATATAAAGCA	This study
	P. malariae-P	FAM-5'-CAATATCTAGTAATGGCTCCATGT-3'-MGB	(14) Modified

3C	VIC-5'-TATTTGCATCGTTACTTAA-3'-MGB	(13) Modified				
6-For	TGTTCATGTGTCCCCTCTTTTTCTG	(13)	*Plasmepsin 4*	*P. ovale*-For	TATCCTCGGAGCACCATTTATGAG	(14)
6-Rev	GGAAGGTAGAATATCAAATACAAGGGATGT	(13)		*P. ovale*-Rev	ACGGCAAAACCGACCCTCTC	(14)
6G	FAM-5'-AATGTTTCTTAGTATGCTTC-3'-MGB	This study		*P. ovale*-P	FAM-5'-ACTTCTCCGTTTTCG-3'-MGB	(14)
6C	VIC-5'-AATGTTTCTTACTATGCTTC-3'-MGB	This study				
			GAPDH	GAPDH-For	CCTCCCGCTTCGCTCTCT	(15, 16)
				GAPDH-Rev	GCTGGCGACGCAAAAGA	(15, 16)
				GAPDH-P	FAM-5'-CCTCCTGTTCGACAGTCAGCCGC -3'-MGB	(15, 16)

For = forward, Rev = reverse, P = probe, Ref. = references, SNP = single nucleotide polymorphism, underline = codon or nucleotide position of mutation, [a] SNP1; *Pf*_01_000130573, SNP2; *Pf*_01_000539044, SNP3; *Pf*_02_000842803, SNP6; *Pf*_06_000145472, SNP7; *Pf*_06_000937750, SNP8; *Pf*_07_000277104

REFERENCES

1. **Straimer J, Gnadig NF, Witkowski B, Amaratunga C, Duru V, Ramadani AP, Dacheux M, Khim N, Zhang L, Lam S, Gregory PD, Urnov FD, Mercereau-Puijalon O, Benoit-Vical F, Fairhurst RM, Menard D, Fidock DA.** 2015. Drug resistance. K13-propeller mutations confer artemisinin resistance in Plasmodium falciparum clinical isolates. Science **347:**428-431.
2. **Ariey F, Witkowski B, Amaratunga C, Beghain J, Langlois AC, Khim N, Kim S, Duru V, Bouchier C, Ma L, Lim P, Leang R, Duong S, Sreng S, Suon S, Chuor CM, Bout DM, Menard S, Rogers WO, Genton B, Fandeur T, Miotto O, Ringwald P, Le Bras J, Berry A, Barale JC, Fairhurst RM, Benoit-Vical F, Mercereau-Puijalon O, Menard D.** 2014. A molecular marker of artemisinin-resistant Plasmodium falciparum malaria. Nature **505:**50-55.
3. **Huang F, Takala-Harrison S, Jacob CG, Liu H, Sun X, Yang H, Nyunt MM, Adams M, Zhou S, Xia Z, Ringwald P, Bustos MD, Tang L, Plowe CV.** 2015. A Single Mutation in K13 Predominates in Southern China and Is Associated With Delayed Clearance of Plasmodium falciparum Following Artemisinin Treatment. J Infect Dis **212:**1629-1635.

4. **Talundzic E, Okoth SA, Congpuong K, Plucinski MM, Morton L, Goldman IF, Kachur PS, Wongsrichanalai C, Satimai W, Barnwell JW, Udhayakumar V.** 2015. Selection and spread of artemisinin-resistant alleles in Thailand prior to the global artemisinin resistance containment campaign. PLoS Pathog **11:**e1004789.

5. **Mishra N, Prajapati SK, Kaitholia K, Bharti RS, Srivastava B, Phookan S, Anvikar AR, Dev V, Sonal GS, Dhariwal AC, White NJ, Valecha N.** 2015. Surveillance of artemisinin resistance in Plasmodium falciparum in India using the kelch13 molecular marker. Antimicrob Agents Chemother **59:**2548-2553.

6. **Taylor SM, Parobek CM, DeConti DK, Kayentao K, Coulibaly SO, Greenwood BM, Tagbor H, Williams J, Bojang K, Njie F, Desai M, Kariuki S, Gutman J, Mathanga DP, Martensson A, Ngasala B, Conrad MD, Rosenthal PJ, Tshefu AK, Moormann AM, Vulule JM, Doumbo OK, Ter Kuile FO, Meshnick SR, Bailey JA, Juliano JJ.** 2015. Absence of putative artemisinin resistance mutations among Plasmodium falciparum in Sub-Saharan Africa: a molecular epidemiologic study. J Infect Dis **211:**680-688.

7. **MalariaGEN.** 2015. P. falciparum Community Project Data https://www.malariagen.net/apps/pf/4.0/. Accessed January 2016.

8. **Wilson PE, Kazadi W, Kamwendo DD, Mwapasa V, Purfield A, Meshnick SR.** 2005. Prevalence of pfcrt mutations in Congolese and Malawian Plasmodium falciparum isolates as determined by a new Taqman assay. Acta Trop **93:**97-106.

9. **Sutherland CJ, Haustein T, Gadalla N, Armstrong M, Doherty JF, Chiodini PL.** 2007. Chloroquine-resistant Plasmodium falciparum infections among UK travellers returning with malaria after chloroquine prophylaxis. J Antimicrob Chemother **59:**1197-1199.

10. **Alker AP, Mwapasa V, Meshnick SR.** 2004. Rapid real-time PCR genotyping of mutations associated with sulfadoxine-pyrimethamine resistance in Plasmodium falciparum. Antimicrob Agents Chemother **48:**2924-2929.

11. **Gadalla NB, Tavera G, Mu J, Kabyemela ER, Fried M, Duffy PE, Sa JM, Wellems TE.** 2015. Prevalence of Plasmodium falciparum anti-malarial resistance-associated polymorphisms in pfcrt, pfmdr1 and pfnhe1 in Muheza, Tanzania, prior to introduction of artemisinin combination therapy. Malar J **14:**129.

12. **Purfield A, Nelson A, Laoboonchai A, Congpuong K, McDaniel P, Miller RS, Welch K, Wongsrichanalai C, Meshnick SR.** 2004. A new method for detection of pfmdr1 mutations in Plasmodium falciparum DNA using real-time PCR. Malar J **3:**9.

13. **Daniels R, Volkman SK, Milner DA, Mahesh N, Neafsey DE, Park DJ, Rosen D, Angelino E, Sabeti PC, Wirth DF, Wiegand RC.** 2008. A general SNP-based molecular barcode for Plasmodium falciparum identification and tracking. Malar J **7:**223.

14. **Reller ME, Chen WH, Dalton J, Lichay MA, Dumler JS.** 2013. Multiplex 5' nuclease quantitative real-time PCR for clinical diagnosis of malaria and species-level identification and epidemiologic evaluation of malaria-causing parasites, including Plasmodium knowlesi. J Clin Microbiol **51:**2931-2938.

15. **Rantala AM, Taylor SM, Trottman PA, Luntamo M, Mbewe B, Maleta K, Kulmala T, Ashorn P, Meshnick SR.** 2010. Comparison of real-time PCR and microscopy for malaria parasite detection in Malawian pregnant women. Malar J **9:**269.

16. **Taylor SM, Juliano JJ, Trottman PA, Griffin JB, Landis SH, Kitsa P, Tshefu AK, Meshnick SR.** 2010. High-throughput pooling and real-time PCR-based strategy for malaria detection. J Clin Microbiol **48:**512-519.

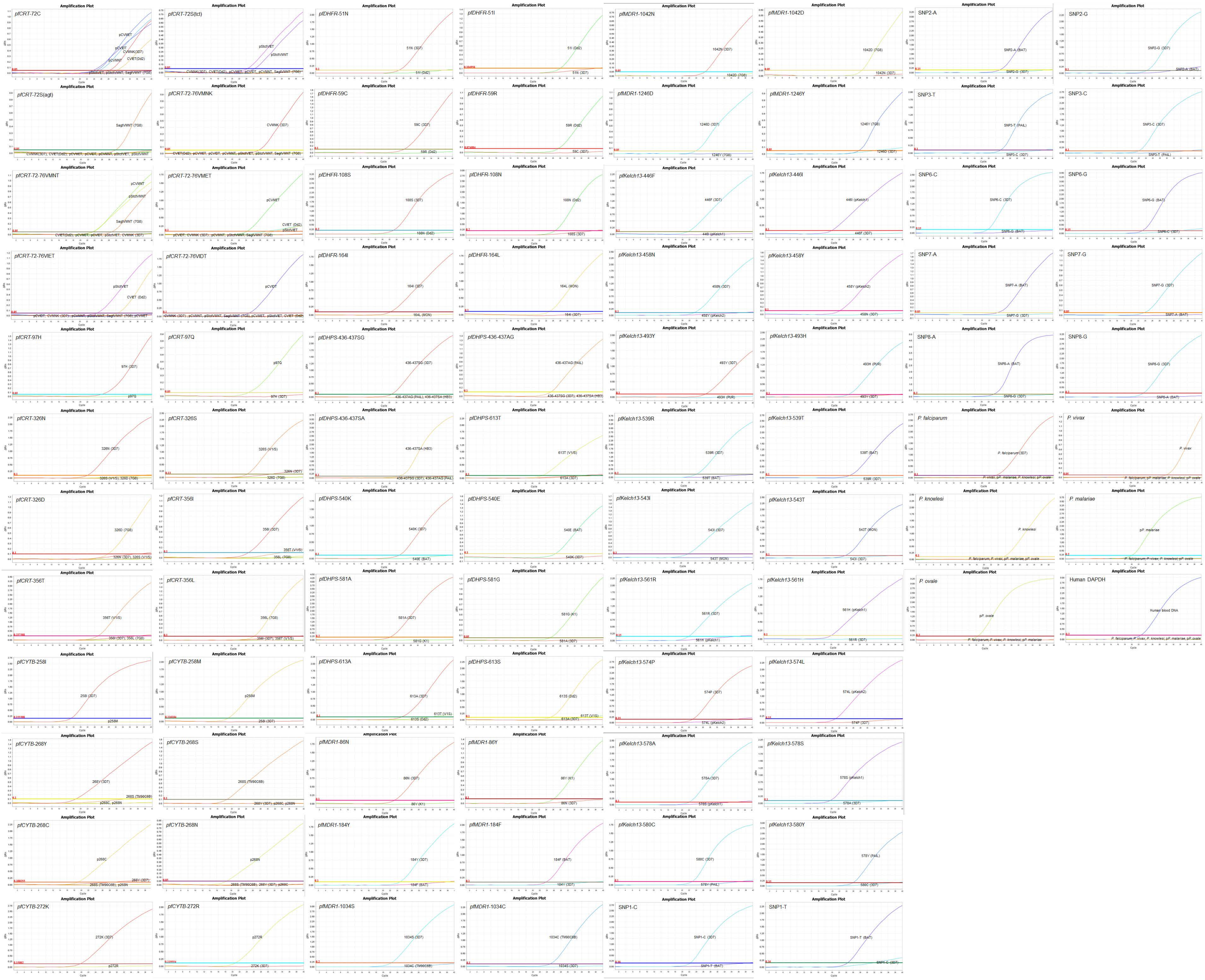
pfCRT-72C
pfCRT-72S(tct)
pfDHFR-51N
pfDHFR-51I
pfMDR1-1042N
pfMDR1-1042D
SNP2-A
SNP2-G
pfCRT-72S(agt)
pfCRT-72-76VMNK
pfDHFR-59C
pfDHFR-59R
pfMDR1-1246D
pfMDR1-1246Y
SNP3-T
SNP3-C
pfCRT-72-76VMNT
pfCRT-72-76VMET
pfDHFR-108S
pfDHFR-108N
pfKelch13-446F
pfKelch13-446I
SNP6-C
SNP6-G
pfCRT-72-76VIET
pfCRT-72-76VIDT
pfDHFR-164I
pfDHFR-164L
pfKelch13-458N
pfKelch13-458Y
SNP7-A
SNP7-G
pfCRT-97H
pfCRT-97Q
pfDHPS-436-437SG
pfDHPS-436-437AG
pfKelch13-493Y
pfKelch13-493H
SNP8-A
SNP8-G
pfCRT-326N
pfCRT-326S
pfDHPS-436-437SA
pfDHPS-613T
pfKelch13-539R
pfKelch13-539T
P. falciparum
P. vivax
pfCRT-326D
pfCRT-356I
pfDHPS-540K
pfDHPS-540E
pfKelch13-543I
pfKelch13-543T
P. knowlesi
P. malariae
pfCRT-356T
pfCRT-356L
pfDHPS-581A
pfDHPS-581G
pfKelch13-561R
pfKelch13-561H
P. ovale
Human DAPDH
pfCYTB-258I
pfCYTB-258M
pfDHPS-613A
pfDHPS-613S
pfKelch13-574P
pfKelch13-574L
pfCYTB-268Y
pfCYTB-268S
pfMDR1-86N
pfMDR1-86Y
pfKelch13-578A
pfKelch13-578S
pfCYTB-268C
pfCYTB-268N
pfMDR1-184Y
pfMDR1-184F
pfKelch13-580C
pfKelch13-580Y
pfCYTB-272K
pfCYTB-272R
pfMDR1-1034S
pfMDR1-1034C
SNP1-C
SNP1-T

Figure S1. Specificity testing of each assay. Specificity testing of the assays was performed on the 384 well plate format. The 35 duplex PCR assays for drug resistance detection were tested with wild-type (3D7) and the well-known mutant parasite lines included Dd2, V1/S, 7G8, TMC90C6B, K1, HB3, PAIL, PUR, MON, and BAT. Synthetic plasmid controls included pCVMET, pCVIDT, pCVMNT, pStctVIET, pStctVMNT, p97Q, p258M, p268C, p268N, p272R, pKelch1 (446I, 561H, 578S), and pKelch2 (458Y, 574L). Each assay shows amplification of only the specific wild-type or mutant. The six duplex assays for SNP genotyping were tested with the well-known major and minor allele for each SNP position including 3D7, BAT, and PAIL; each assay shows amplification of only the specific major or minor allele; SNP1 refers to Pf_01_000130573, SNP2 refers to Pf_01_000539044, SNP3 refers to Pf_02_000842803, SNP6 refers to Pf_06_000145472, SNP7 refers to Pf_06_000937750, and SNP8 refers to Pf_07_000277104 position. Five singleplex assays for species identification were tested with P. falciparum 3D7, P. vivax, P. knowlesi, and synthetic plasmid control for P. ovale and P. malariae; each assay shows amplification of only the specific species. The hGAPDH assay was tested against human blood DNA and the five Plasmodium species; only human blood DNA was amplified.

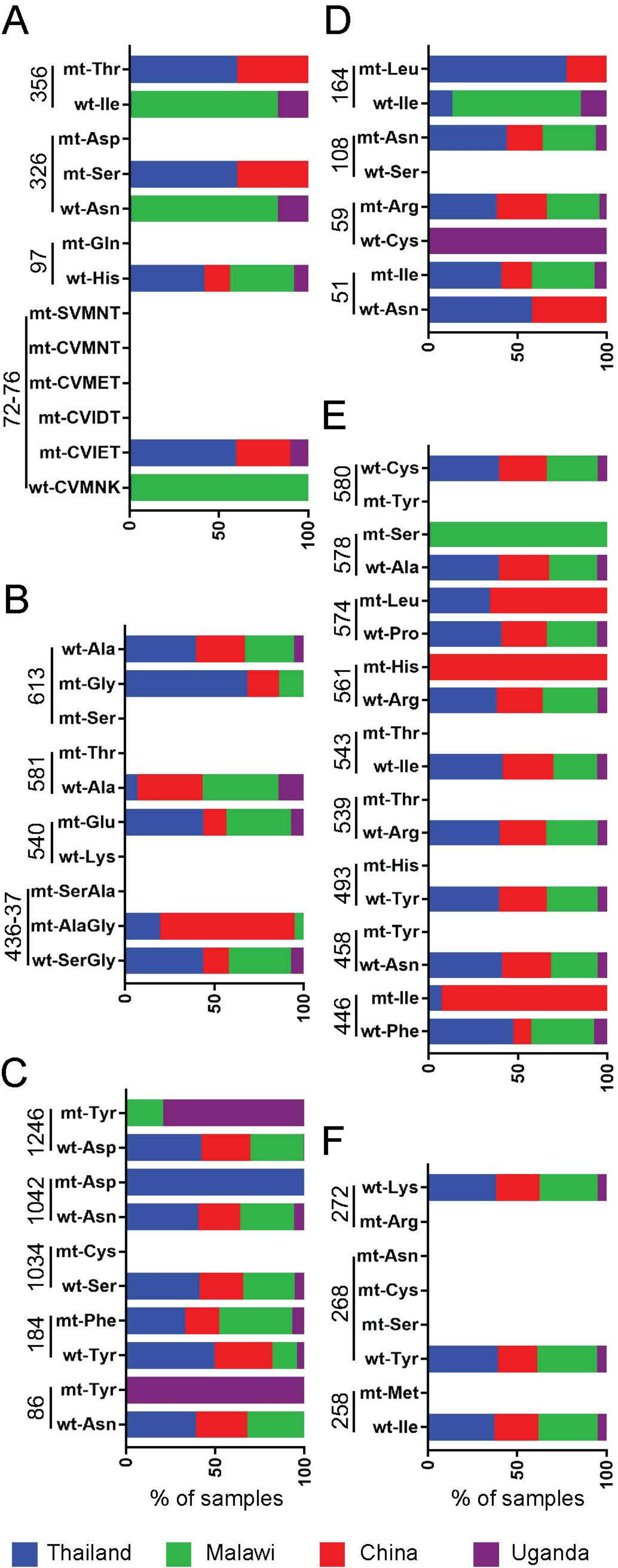

A
356
mt-Thr
wt-Ile
326
mt-Asp
mt-Ser
wt-Asn
97
mt-Gln
wt-His
72-76
mt-SVMNT
mt-CVMNT
mt-CVMET
mt-CVIDT
mt-CVIET
wt-CVMNK
0
50
100
B
613
wt-Ala
mt-Gly
mt-Ser
581
mt-Thr
wt-Ala
540
mt-Glu
wt-Lys
436-37
mt-SerAla
mt-AlaGly
wt-SerGly
0
50
100
C
1246
mt-Tyr
wt-Asp
1042
mt-Asp
wt-Asn
1034
mt-Cys
wt-Ser
184
mt-Phe
wt-Tyr
86
mt-Tyr
wt-Asn
0
50
100
% of samples
D
164
mt-Leu
wt-Ile
108
mt-Asn
wt-Ser
59
mt-Arg
wt-Cys
51
mt-Ile
wt-Asn
0
50
100
E
580
wt-Cys
mt-Tyr
578
mt-Ser
wt-Ala
574
mt-Leu
wt-Pro
561
mt-His
wt-Arg
543
mt-Thr
wt-Ile
539
mt-Thr
wt-Arg
493
mt-His
wt-Tyr
458
mt-Tyr
wt-Asn
446
mt-Ile
wt-Phe
0
50
100
F
272
wt-Lys
mt-Arg
268
mt-Asn
mt-Cys
mt-Ser
wt-Tyr
258
mt-Met
wt-Ile
0
50
100
% of samples
Thailand
Malawi
China
Uganda

Figure S2. Allele distribution of clinical *P. falciparum* samples as determined by malaria-TAC. The percent of total positive samples for either wild-type (wt) or mutant (mt) versions of each codon of antimalarial resistance-associated genes (**A**. *pfCRT*, **B**. *pfDHPS*, **C**. *pfMDR1*, **D**.*pfDHFR*, **E**. *pfKELCH13*, **F**. *pfCYTB*). Sample origins are denoted by color: Thailand (blue), China (red), Malawi (green), and Uganda (purple). Percent of samples was calculated by dividing the number of samples with the specific allele for each country by the total samples with that allele and multiplying by 100. The amino acid of *pfCRT* at codon 72-76 included Cys (C)/Ser (S), Val (V), Met (M)/Ile (I), Asn (N)/Glu (E)/Asp (D), Lys (K)/Thr (T).

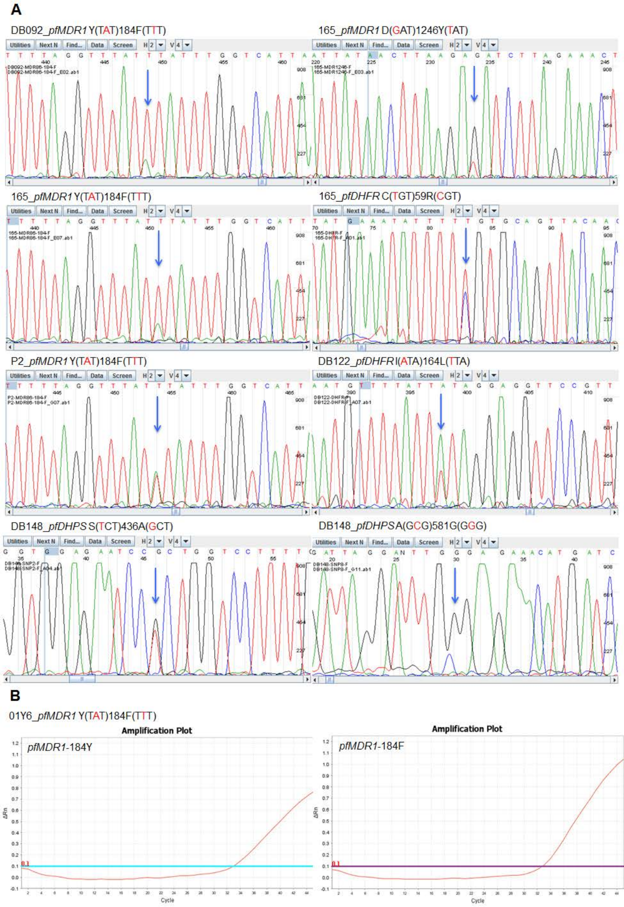
A
DB092_pfMDR1 Y(TAT)184F(TTT)
165_pfMDR1 D(GAT)1246Y(TAT)
165_pfMDR1 Y(TAT)184F(TTT)
165_pfDHFR C(TGT)59R(CGT)
P2_pfMDR1 Y(TAT)184F(TTT)
DB122_pfDHFR I(ATA)164L(TTA)
DB148_pfDHPS S(TCT)436A(GCT)
DB148_pfDHPS A(GCG)581G(GGG)
B
01Y6_pfMDR1 Y(TAT)184F(TTT)
Amplification Plot
pfMDR1-184Y
Amplification Plot
pfMDR1-184F
ΔRn
Cycle

Figure S3. Hetero-resistance as determined by the malaria-TAC. 7/85 samples were detected as mixed alleles by the malaria-TAC and then sequenced confirmed. **A**. Sanger sequencing shows mixed nucleotide (blue arrow) indicating mixed wild-type and mutant alleles for 5 samples DB092, 165, P2, DB122, and DB148 (DB133 is shown in Fig. 2). **B**. TaqMan-probe based amplification plots are shown for the nonsuccessful sequencing of sample 01Y6 to illustrate the true mix allele detected by TAC.

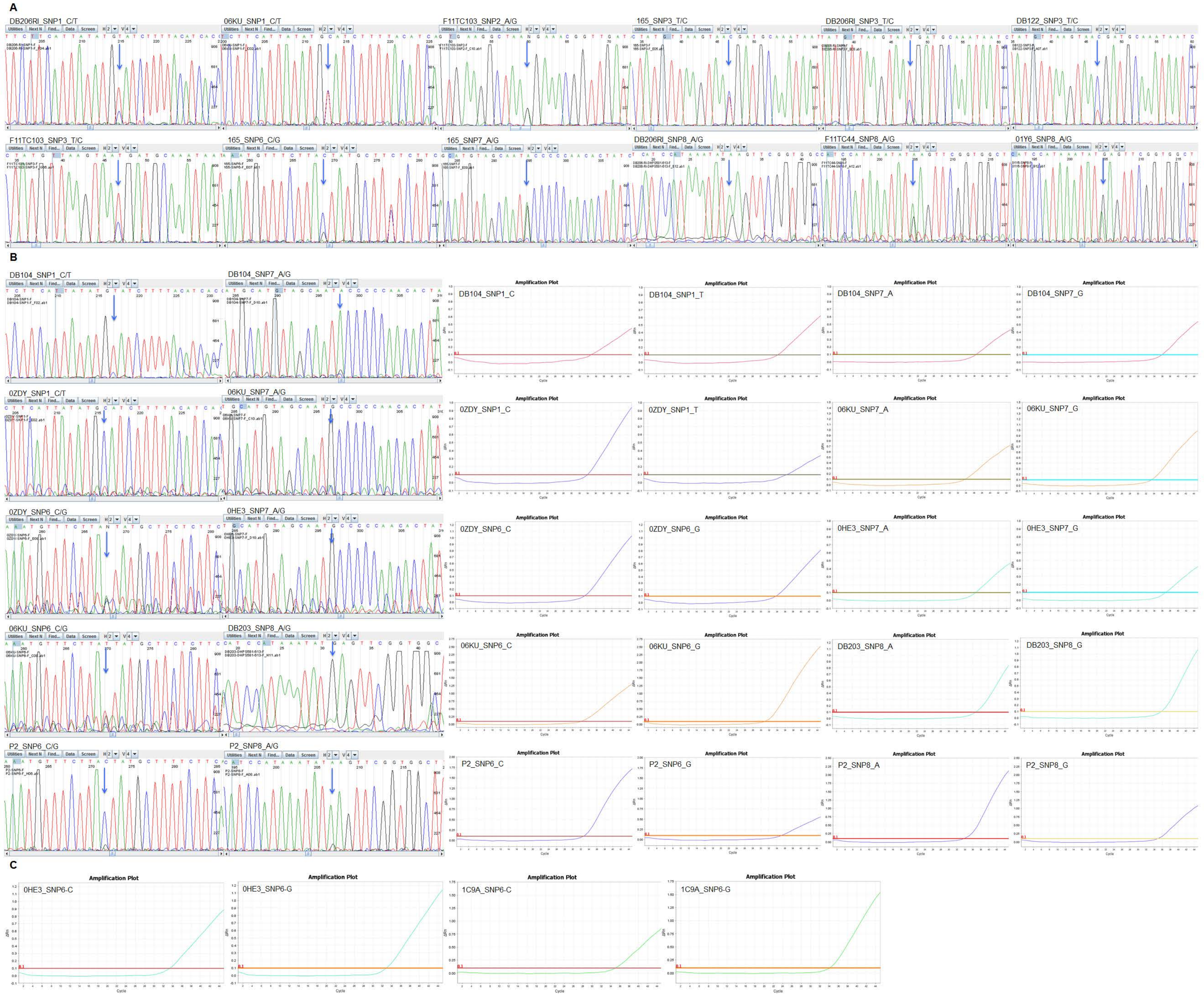
A
DB206RI_SNP1_C/T
06KU_SNP1_C/T
F11TC103_SNP2_A/G
165_SNP3_T/C
DB206RI_SNP3_T/C
DB122_SNP3_T/C
F11TC103_SNP3_T/C
165_SNP6_C/G
165_SNP7_A/G
DB206RI_SNP8_A/G
F11TC44_SNP8_A/G
01Y6_SNP8_A/G
B
DB104_SNP1_C/T
DB104_SNP7_A/G
DB104_SNP1_C
DB104_SNP1_T
DB104_SNP7_A
DB104_SNP7_G
0ZDY_SNP1_C/T
06KU_SNP7_A/G
0ZDY_SNP1_C
0ZDY_SNP1_T
06KU_SNP7_A
06KU_SNP7_G
0ZDY_SNP6_C/G
0HE3_SNP7_A/G
0ZDY_SNP6_C
0ZDY_SNP6_G
0HE3_SNP7_A
0HE3_SNP7_G
06KU_SNP6_C/G
DB203_SNP8_A/G
06KU_SNP6_C
06KU_SNP6_G
DB203_SNP8_A
DB203_SNP8_G
P2_SNP6_C/G
P2_SNP8_A/G
P2_SNP6_C
P2_SNP6_G
P2_SNP8_A
P2_SNP8_G
Amplification Plot
Cycle
C
0HE3_SNP6-C
0HE3_SNP6-G
1C9A_SNP6-C
1C9A_SNP6-G

Figure S4. Mixed infection as determined by the malaria-TAC. The samples were detected as mixed alleles by the malaria-TAC and then sequenced confirmed. **A**. Sanger sequencing shows a clear mixed nucleotide (blue arrow) indicated mixed major and minor allele. **B**. Sanger sequencing shows an unclear mixed nucleotide (blue arrow in the left panel). TaqMan-probe based amplification plots are shown to illustrate the true mixed allele detected by TAC (right panel). **C**. TaqMan-probe based amplification plots are shown for the nonsuccessful sequencing of sample 0HE3 and 1C9A to illustrate the true mixed allele detected by TAC. SNP1 refers to Pf_01_000130573, SNP2 refers to Pf_01_000539044, SNP3 refers to Pf_02_000842803, SNP6 refers to Pf_06_000145472, SNP7 refers to Pf_06_000937750, and SNP8 refers to Pf_07_000277104 position.
